# Advances and Prospects of Chiral Plasmonic Nanomaterials in Emerging Applications

**DOI:** 10.3390/nano16171099

**Published:** 2026-09-01

**Authors:** Panangattukara Prabhakaran Praveen Kumar

**Affiliations:** Department of Biomedical Engineering, Institute for Quantitative Health Science and Engineering, Michigan State University, East Lansing, MI 48824, USA; pananga1@msu.edu or p4praveen.18@gmail.com

**Keywords:** chirality, chiral nanoparticles, biosensing, enantioselective detection, bioimaging, therapy

## Abstract

Chiral plasmonic nanostructures have emerged as a distinctive class of optical materials that combine nanoscale structural asymmetry with strong light–matter interactions, enabling pronounced and tunable chiroptical responses. Advances in structural engineering and plasmonic coupling have enabled diverse architectures with potential applications in enantioselective sensing, asymmetric catalysis, bioimaging, and therapeutic technologies. However, their broader development remains constrained by several key challenges, including precise control over nanoscale chirality, batch-to-batch structural reproducibility, incomplete understanding of structure–chiroptical property relationships, scalable fabrication, and stability under practical and biological conditions. This review systematically examines the major structural architectures of chiral plasmonic nanomaterials and the mechanisms responsible for chirality generation, including ligand-induced, intrinsic geometric, and assembly induced chirality. Particular emphasis is placed on correlating structural design, plasmonic coupling, and chiroptical properties with functional performance. Recent advances in enantioselective recognition and sensing, catalytic transformations, and biomedical applications are critically discussed, together with their current limitations. Finally, emerging design strategies and future opportunities for improving structural precision, reproducibility, scalability, and practical translation are highlighted, providing a framework for the rational development of next-generation chiral plasmonic nanomaterials.

## 1. Introduction

Chirality is a fundamental characteristic of matter that is widely observed in both natural and synthetic systems [1,2,3]. A structure is considered chiral when it cannot be superimposed onto its mirror image. This phenomenon plays a central role in biological processes, as many essential biomolecules including DNA, proteins, amino acids, and polysaccharides possess inherent chirality. Molecular handedness governs numerous biological interactions, influencing molecular recognition, catalytic activity, and physiological function [4,5]. Consequently, understanding and controlling chirality have become increasingly important across chemistry, materials science, and biomedicine.

The emergence of nanotechnology has expanded the significance of chirality beyond molecular systems to nanoscale materials [6,7]. Compared with their molecular counterparts, chiral nanomaterials often exhibit amplified chiroptical responses arising from their unique size- and shape-dependent optical properties. Advances in nanoparticle synthesis have enabled precise control over composition, morphology, and hierarchical organization, allowing the fabrication of chiral nanostructures with tunable optical characteristics [8,9,10]. Chiral behavior has now been demonstrated in a wide range of inorganic nanomaterials, including noble metals [11,12], semiconductors [13,14], metal oxides [15,16], and their assembled architectures [17,18]. These materials display strong circular dichroism and related chiroptical phenomena, creating opportunities in enantiomeric recognition [19,20], asymmetric catalysis [21,22], biosensing [23,24], molecular imaging [25,26], polarization optics [27,28], and photonic technologies [29,30].

Among the various inorganic nanomaterials, noble metal nanostructures have attracted particular attention because of their exceptional plasmonic properties [31]. Gold, silver, platinum, palladium, rhodium, ruthenium, iridium, and osmium support localized surface plasmon resonance (LSPR), which originates from the collective oscillation of conduction electrons upon interaction with incident light. The wavelength, intensity, and spectral characteristics of LSPR are highly sensitive to nanoparticle size, geometry, composition, and the surrounding dielectric environment. This tunability provides a powerful platform for engineering optical responses with high sensitivity and selectivity. The strong plasmonic characteristics of noble metal nanomaterials have enabled numerous applications in analytical science and biomedicine [32]. Local electromagnetic field enhancement associated with LSPR significantly improves optical signal generation, facilitating highly sensitive and label-free detection of biomolecular interactions while preserving the native state of biological targets. In addition, efficient absorption of near-infrared light allows these nanomaterials to convert optical energy into heat with remarkable efficiency, making them attractive candidates for photothermal therapy, multimodal imaging, and image-guided therapeutic interventions [33,34]. The combination of plasmonic enhancement and structural chirality further broadens their potential by enabling polarization-dependent optical responses and improved interactions with chiral biological systems [35].

This review provides a comprehensive overview of the design principles, chiroptical properties, and biomedical applications of chiral noble metal nanomaterials. We first discuss the fundamental mechanisms responsible for their optical activity and then systematically classify the origins of chirality into three structural levels. The first category, atomic-level chirality, arises from chiral ligands, asymmetric surface coordination, or intrinsic stereochemical arrangements within metal nanoclusters, where ligand–metal orbital interactions generate chiroptical activity. The second, nanoscale geometric chirality, originates from asymmetric nanoparticle morphologies, including helical, twisted, and anisotropically grown structures, which exhibit pronounced plasmonic circular dichroism and circularly polarized luminescence. The third category, hierarchical assembly induced chirality, emerges from the organization of nanoparticles into larger chiral superstructures directed by templates such as DNA origami, chiral polymers, peptides, or cellulose nanocrystals, where collective plasmonic coupling produces enhanced chiral optical responses.

Unlike previous reviews that have largely addressed the synthesis, assembly, or chiroptical characteristics of chiral plasmonic nanomaterials as separate themes, this review adopts an integrated chirality origin–structural design–chiroptical property–application framework. Specifically, we correlate chirality generated at the atomic, nanoscale geometric, and hierarchical assembly levels with the corresponding plasmonic responses and functional performance. This structure enables direct comparison of how different chirality-generation and fabrication strategies influence optical activity and, ultimately, their suitability for specific applications. Building on this framework, we critically examine recent advances in biosensing, enantioselective recognition and separation, bioimaging, photothermal therapy, cancer theranostics, and catalytic applications. Finally, we identify key challenges related to structural control, reproducibility, mechanistic understanding, biocompatibility, and scalability, and discuss emerging opportunities for the rational design and translation of next-generation chiral plasmonic nanomaterials.

## 2. Optical Properties of Chiral NPs

### 2.1. Localized Surface Plasmon Resonance

**Figure 1:**
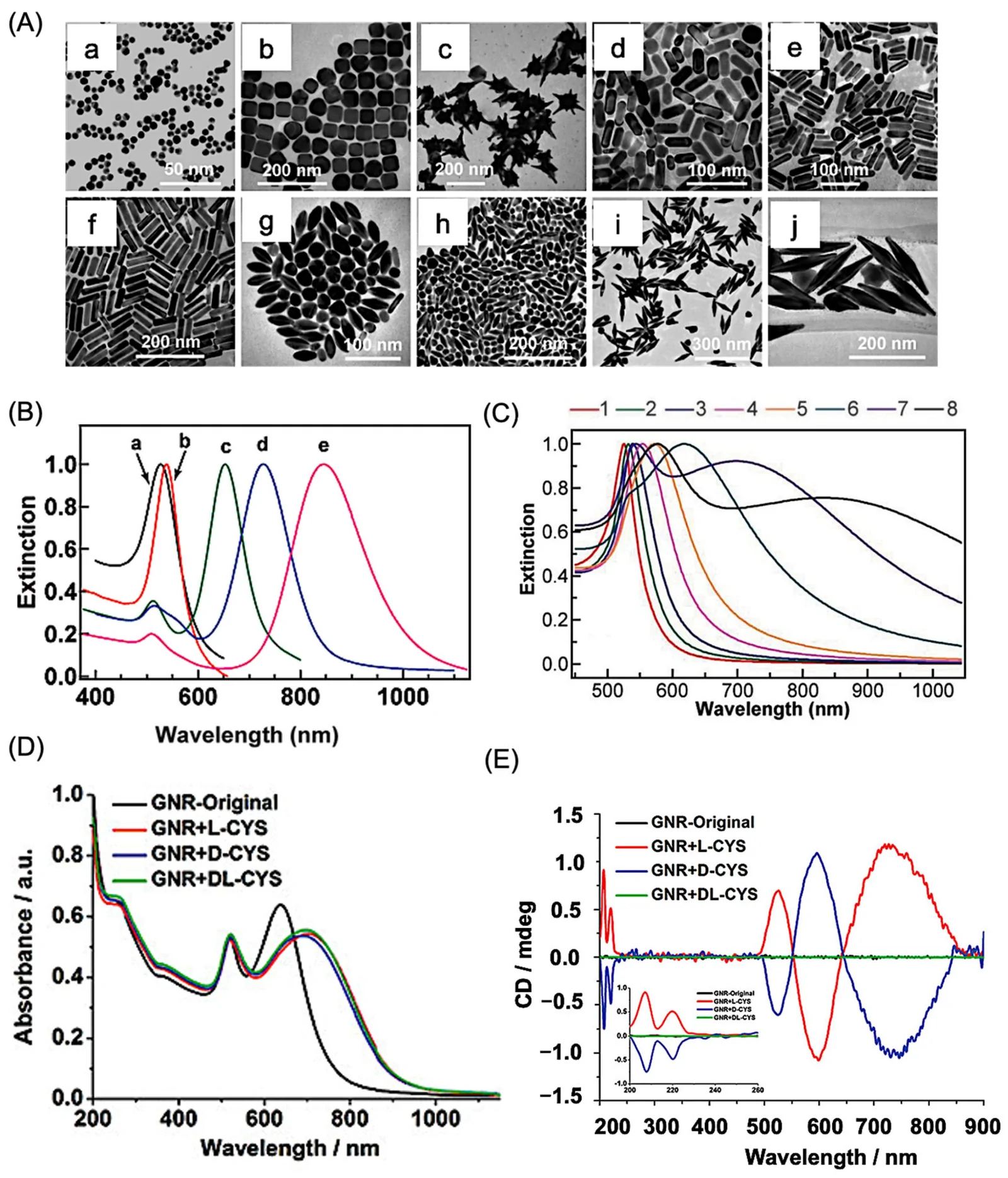
Structural diversity and optical characteristics of plasmonic gold nanostructures. Panel (**A**) TEM images of (**a**) nanospheres, (**b**) nanocubes, (**c**) nanobranches, (**d**–**f**) nanorods, and (**g**–**j**) nanobipyramids with varying aspect ratios. (**B**) Morphology-dependent extinction spectra of gold nanostructures. Spectra a−e correspond to nanospheres, nanocubes, and nanorods with aspect ratios of 2.4 ± 0.3, 3.4 ± 0.5, and 4.6 ± 0.8, respectively. (**C**) Experimental and Mie theory-simulated extinction spectra showing size-dependent LSPR of gold nanospheres. The samples 1 to 8 are the Au NS samples with average diameters of 24 ± 2 nm, 54 ± 2 nm, 69 ± 3 nm, 88 ± 4 nm, 103 ± 6 nm, 137 ± 9 nm, 177 ± 14 nm, and 221 ± 16 nm, respectively. (**D**) UV–Vis–NIR spectra of individual and cysteine-induced assembled gold nanorods. (**E**) CD spectra of pristine and cysteine-assembled gold nanorods, demonstrating chirality transfer and plasmonic amplification. (**A**,**B**) reproduced with permission from [36]; Copyright 2008, American Chemical Society. Panel (**C**) reproduced with permission from [37]; Copyright 2014, Wiley Publishers. Panel (**D**,**E**) reproduced with permission from [38]; Copyright 2012, American Chemical Society.

Localized surface plasmon resonance (LSPR) is a defining optical property of noble metal nanoparticles, arising from the collective oscillation of conduction electrons under incident electromagnetic radiation. This phenomenon produces characteristic absorption bands in the UV–visible–NIR region, whose spectral position and intensity are governed by nanoparticle size, shape, composition, and the surrounding dielectric environment. Consequently, precise control over these structural parameters enables systematic tuning of plasmonic responses (Figure 1A,B) [36].

Advances in nanofabrication have enabled the synthesis of highly monodisperse noble metal nanostructures with diverse morphologies, including nanospheres, nanorods, nanosheets, nanocones, cubes, and octahedra, each exhibiting distinct LSPR characteristics [37]. Among these, gold nanoparticles are the most extensively investigated for biomedical applications. Increasing particle size shifts the LSPR band toward longer wavelengths while enhancing plasmonic intensity; for example, the LSPR peak of gold nanoparticles red shifts from approximately 521 to 806 nm as the particle diameter increases from 24 to 221 nm. Interactions between noble metal nanoparticles and chiral molecules generate chiroptical responses at the LSPR wavelength, with signal intensity generally increasing as particle size increases (Figure 1C) [37]. Nanoparticle morphology provides an additional level of control over plasmonic behavior. In gold nanorods (GNRs), increasing the aspect ratio progressively red-shifts the longitudinal LSPR into the near-infrared region while simultaneously strengthening the circular dichroism (CD) response. As a result, the CD signal can be tuned from approximately 550 nm to beyond 900 nm (Figure 1D,E) [38]. This enhancement has been attributed to two synergistic effects: stronger longitudinal plasmon oscillations that promote more pronounced chiral currents within individual nanorods and enhanced near-field coupling between adjacent GNRs, which creates intense electromagnetic “hot spots” that further amplify the chiroptical response.

### 2.2. Circular Dichroism Properties

Chirality is a fundamental structural property in which an object or molecule cannot be superimposed on its mirror image. This asymmetry gives rise to unique optical phenomena, including circular dichroism (CD) and circularly polarized luminescence (CPL)**,** which are widely employed to characterize chiral materials [39]. Among these techniques, CD spectroscopy is the most used method for probing molecular and nanoscale chirality. CD measures the differential absorption of left- and right-handed circularly polarized light (LCP and RCP), which can be expressed as

ΔA = A_L_ − A_R_

where A_L_ and A_R_ denote the absorbance of left- and right-handed circularly polarized light, respectively.

To quantitatively evaluate the intrinsic chiral response of a material, the dissymmetry factor (g-factor) is commonly employed:

g = 2 (A_L_ − A_R_)/ (A_L_ + A_R)_

Unlike the raw CD signal, the g-factor normalizes the differential absorption to the average absorbance, eliminating the influence of sample concentration and optical path length. Consequently, it provides a direct measure of the inherent chiral asymmetry of a material. The magnitude of the g-factor varies significantly among different classes of chiral systems. Small organic molecules, including amino acids and peptides, typically exhibit relatively weak chiroptical responses, with g-factors in the range of 10^−4^–10^−3^ [40]. However, hierarchical self-assembly into supramolecular architectures such as helical fibers or twisted ribbons can substantially amplify the chiral response, increasing g-factors to approximately 10^−2^ [41]. Similarly, inorganic nanomaterials functionalized with chiral ligands generally exhibit g-factors ranging from 10^−4^ to 10^−2^, whereas plasmonic nanoparticle assemblies possessing collective chiral arrangements frequently achieve values on the order of 10^−2^ due to enhanced electromagnetic coupling [42].

Chiral emission is characterized by the luminescence dissymmetry factor g_(lum)_ [43], which describes the difference in left- and right-handed circularly polarized emission:

g_(lum)_ = 2 (I_L_ − I_R_)/(I_L_ + I_R_)

where I_L_ and I_R_ represent the emission intensities of left- and right-handed circularly polarized light, respectively. Although g_(lum)_ provides a quantitative measure of CPL performance, it should be interpreted together with the photoluminescence quantum yield [44]. A high $g_{lum}$ alone does not necessarily indicate an efficient chiral emitter, particularly for artificial inorganic chiral nanostructures where emission efficiency is equally critical for practical applications.

### 2.3. Electrodynamic Basis of Chiroptical Responses

The strong chiroptical activity of plasmonic nanostructures can be understood from the electrodynamic coupling between their induced electric and magnetic responses [45]. At the dipolar level, the optical response of a chiral nanostructure can be represented by induced electric (p) and magnetic (m) dipole moments:

p = α_ee_(ω) · E + α_em_(ω) · H.

m = α_me_(ω) · E + α_mm_(ω) · H.

where E and H are the incident electric and magnetic fields, α_ee_ and α_mm_ describe the electric magnetic polarizabilities, respectively, and the cross-polarizability terms α_em_ and α_me_ describe magnetoelectric coupling [46]. In achiral structures, such cross-coupling is generally absent or weak, whereas structural asymmetry in chiral plasmonic systems enables coupling between electric and magnetic modes. The relative amplitude and phase of these induced modes determine their interference and consequently the differential interaction with left- and right-circularly polarized light. This provides an electrodynamic basis for the experimentally observed CD response and optical dissymmetry of chiral plasmonic nanostructures [47].

Although the dipole approximation provides an intuitive description for relatively small and simple nanoparticles, the optical response of larger or structurally complex chiral architectures often involves substantial contributions from higher-order multipolar modes [48]. Their electromagnetic response can therefore be considered as a combination of electric dipole (p), magnetic dipole (m), electric quadrupole (Qe), magnetic quadrupole (Qm), and higher-order terms. The excitation and interference of these modes are strongly dependent on nanoparticle dimensions, morphology, degree of structural asymmetry, and relative orientation of individual plasmonic components. In helicoidal, twisted, and hierarchically assembled nanostructures, interference between dipolar and multipolar resonances can substantially alter the magnitude, sign, and spectral position of CD signals. Thus, structural engineering of plasmonic nanoparticles provides a means of controlling not only the LSPR position but also the relative contribution and coupling of electromagnetic multipoles, enabling stronger and spectrally tunable chiroptical responses.

An important consequence of plasmonic coupling is the formation of highly confined electromagnetic regions known as chiral hot spots, particularly near asymmetric surface features and within nanoscale gaps between coupled nanoparticles [49]. The handedness of the local electromagnetic field can be quantitatively described by the optical chirality density. For monochromatic electromagnetic fields, it can be expressed as

C = −(ε_0_ω/2)Im(*E*^∗^⋅*B*)

where ε_0_ is the vacuum permittivity, ω is the angular frequency, and *E* and *B* represent the complex electric and magnetic fields, respectively. Appropriately engineered plasmonic nanostructures can generate local fields with optical chirality exceeding that of conventional circularly polarized light, commonly referred to as superchiral fields [50,51]. Such fields can strongly enhance the differential interaction of opposite molecular enantiomers with light. Importantly, the resulting sensing enhancement depends not only on the magnitude of the electromagnetic field but also on the spatial distribution of optical chirality and its overlap with the target molecule. Engineering nanogaps, asymmetric facets, and coupled plasmonic architectures can therefore generate localized chiral hot spots that provide a physical basis for ultrasensitive enantiomeric recognition and chiral sensing.

Beyond these linear optical effects, plasmonic nanostructures can also support pronounced nonlinear chiroptical responses. The intense local electromagnetic fields generated near plasmonic resonances can enhance nonlinear processes such as second-harmonic generation (SHG) [52,53], third-harmonic generation (THG) [54], and multiphoton interactions [55]. Because nonlinear polarization is highly sensitive to structural symmetry and local field distributions, chiral nanostructures can exhibit handedness-dependent nonlinear responses and enhanced contrast between opposite enantiomorphic structures. Such nonlinear chiroptical phenomena provide additional sensitivity to nanoscale structural asymmetry and offer opportunities for nonlinear chiral spectroscopy, polarization-selective sensing, optical switching, and nanophotonic applications. Collectively, electric–magnetic dipole coupling, higher-order multipolar interference, localized chiral hot spots, superchiral fields, and nonlinear optical interactions provide the fundamental electrodynamic framework linking structural chirality to the enhanced optical responses of plasmonic nanomaterials [47].

## 3. Origin of Plasmonic Chirality

Chirality describes the geometric property of an object that cannot be superimposed onto its mirror image. Although molecular chirality commonly arises from asymmetric carbon centers, it can also originate from helical molecular conformations or supramolecular arrangements. In plasmonic nanomaterials, chirality extends beyond individual molecules and can emerge through interactions between metallic nanostructures and chiral ligands, ordered nanoparticle assemblies, or intrinsically asymmetric nanostructures. These nanoscale architectures generate much stronger chiroptical responses than most isolated organic molecules because of the unique optical properties associated with localized surface plasmon resonance (LSPR).

**Figure 2:**
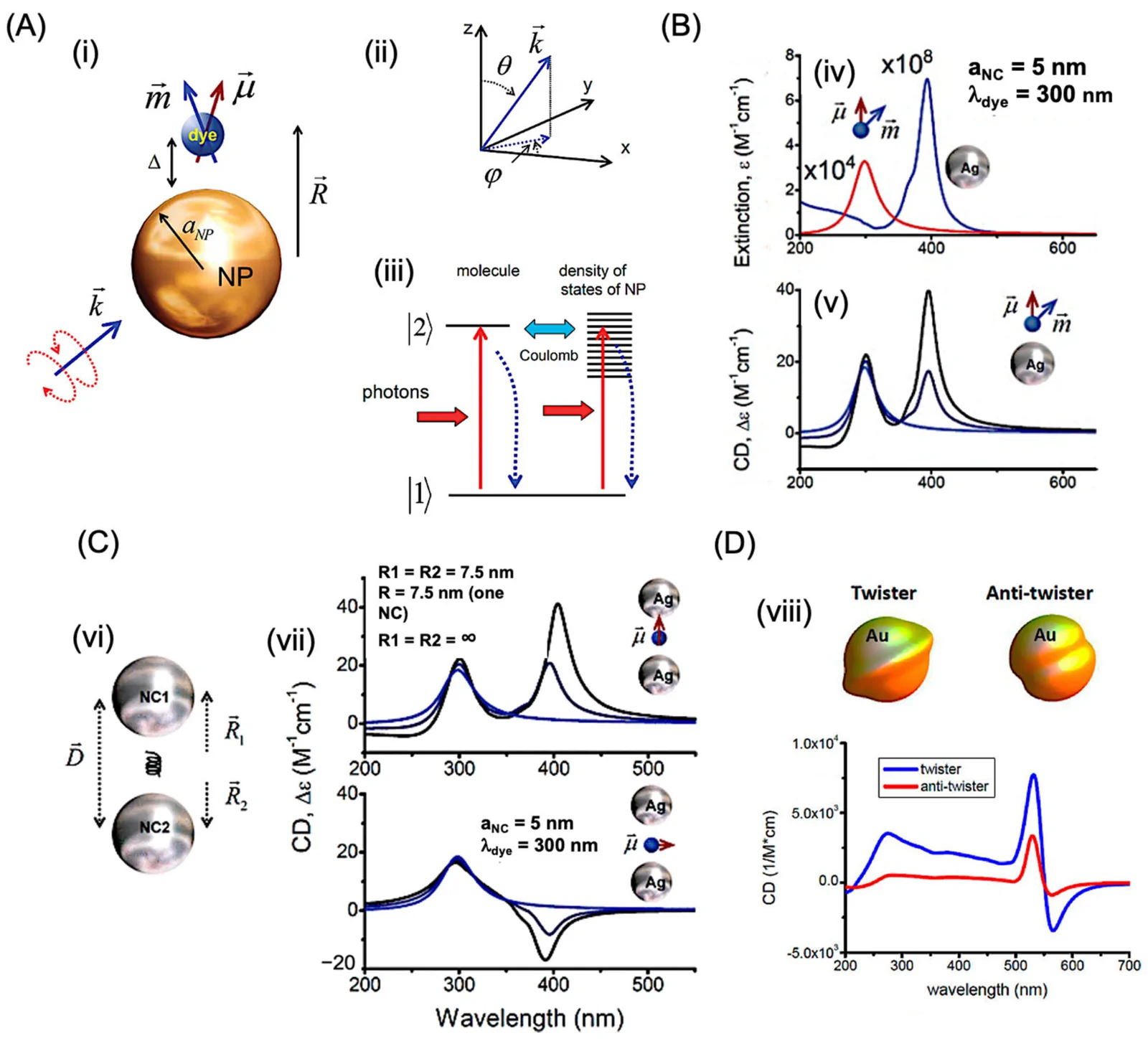
Representative mechanisms underlying plasmon-enhanced chirality. (**A**) Nanoparticle–molecule geometry (**i**) and light-induced electronic interactions (**ii**,**iii**). (**B**) Simulated extinction (**iv**) and CD spectra (**v**) showing the effect of nanoparticle–molecule separation. (**C**) Chiral molecule within a plasmonic dimer hotspot (**vi**) and corresponding CD enhancement (**vii**). (**D**) Oppositely handed chiral gold nanoparticles and their calculated CD responses (**viii**), illustrating geometry-dependent optical activity. Panel (**A**) is reproduced with permission from [56]; Copyright 2010, American Chemical Society. Panels (**B**,**C**) reproduced with permission from [57]; Copyright 2011, American Chemical Society. Panel (**D**) reproduced with permission from [58]; Copyright 2012, American Chemical Society.

Upon light irradiation, conduction electrons within metallic nanoparticles undergo collective oscillations, producing localized surface plasmon resonances that concentrate electromagnetic energy near the nanoparticle surface. These enhanced near fields dramatically strengthen light–matter interactions and amplify the optical response of nearby chiral molecules. As illustrated in Figure 2A, a chiral molecule positioned close to a plasmonic nanoparticle experiences strong electromagnetic coupling, resulting in an enhanced circular dichroism (CD) response compared with the molecule alone [56]. The intensity of this enhancement depends on several factors, including nanoparticle composition, size, morphology, and, particularly, the separation distance between the molecule and the nanoparticle surface. The enhancement becomes even more pronounced when chiral molecules are located within plasmonic hotspots, which are highly confined electromagnetic fields generated in the nanogaps between adjacent nanoparticles. As schematically shown in Figure 2B, nanoparticle dimers create intense, localized fields that significantly amplify the interaction between circularly polarized light and the chiral molecule [57]. Consequently, hotspot engineering has become an effective strategy for improving the sensitivity of plasmonic chiral sensors and spectroscopic platforms.

Besides molecule-mediated chirality, plasmonic nanoparticles can also acquire optical activity through their spatial organization. Self-assembly of metallic nanoparticles into left- or right-handed helices, twisted chains, or other asymmetric architectures generates collective plasmonic coupling that produces characteristic CD spectra. Figure 2C conceptually illustrates this mechanism, where the three-dimensional arrangement of nanoparticles dictates the handedness and magnitude of the optical response [57]. Unlike molecular chirality, these collective plasmonic modes can be readily tuned by controlling interparticle spacing, assembly geometry, and nanoparticle dimensions. An alternative approach involves fabricating nanoparticles with intrinsically chiral morphologies. Twisted, helical, or asymmetrically grown metallic nanostructures exhibit inherent optical activity without requiring chiral molecular ligands or templates. As represented in Figure 2D, the geometric asymmetry of these nanoparticles directly determines their interaction with circularly polarized light, leading to strong and tunable chiroptical signals [58]. Advances in synthetic methodologies have enabled precise control over nanoparticle morphology, allowing the design of chiral plasmonic systems with tailored optical properties for biosensing, imaging, catalysis, and photonic applications [47].

Because the optical response of complex plasmonic architectures is governed by intricate electromagnetic interactions, computational modeling has become an indispensable tool for understanding and optimizing chiral nanostructures [59]. Numerical approaches, including finite-difference time-domain (FDTD), finite-element method (FEM), boundary element method (BEM), and discrete dipole approximation (DDA), are widely employed to predict electromagnetic field distributions, plasmonic coupling, and CD spectra [60,61]. These simulations complement experimental investigations by revealing how structural parameters influence chiroptical activity and by guiding the rational design of next-generation plasmonic nanomaterials. Overall, plasmonic chirality originates primarily from three complementary mechanisms: (i) electromagnetic coupling between plasmonic nanoparticles and chiral molecules, (ii) collective plasmonic interactions within chiral nanoparticle assemblies, and (iii) intrinsically chiral nanoparticle geometries [47]. Together, these mechanisms provide versatile routes for amplifying chiroptical responses and have established plasmonic nanostructures as promising platforms for molecular recognition, biosensing, biomedical imaging, and light-mediated therapeutic applications.

## 4. Chiral Plasmonic Nanoparticles Design Strategies

Chiral plasmonic nanoparticles (NPs), particularly those composed of noble metals such as gold (Au), silver (Ag), and copper (Cu), have emerged as an important class of nanomaterials owing to their unique optical responses arising from LSPR [25,62]. Unlike conventional plasmonic nanomaterials, chiral plasmonic nanoparticles interact differently with left- and right-handed circularly polarized light, giving rise to measurable circular dichroism (CD) signals. This differential optical response results from asymmetric light–matter interactions and serves as the primary signature of plasmonic chirality. Owing to strong electromagnetic field confinement at the nanoparticle surface, plasmonic systems often exhibit chiroptical responses that are significantly stronger than those observed in small chiral molecules, making them attractive platforms for optical sensing, imaging, and photonic applications.

The origin of plasmonic chirality has been extensively investigated through theoretical and experimental studies. Electromagnetic simulations have demonstrated that adsorption of chiral molecules onto plasmonic nanoparticles induces asymmetric electric fields around the metal surface. These asymmetric fields generate chiral plasmonic currents that modify the localized electromagnetic environment, ultimately producing plasmon-enhanced circular dichroism. The resulting CD intensity is closely associated with the strength of the localized plasmon resonance and is highly sensitive to the orientation, spatial arrangement, and coupling of chiral dipoles near the nanoparticle surface. Consequently, structural modifications that strengthen plasmonic coupling generally lead to enhanced chiroptical responses. Among the various structural parameters, particle geometry plays a dominant role in determining plasmonic chirality. Helical or twisted nanoparticle architectures produce stronger optical dissymmetry than symmetric nanostructures because their three-dimensional asymmetry promotes efficient coupling with circularly polarized light. Increasing the degree of helicity or reducing the spacing between neighboring structural features enhances electromagnetic coupling, leading to stronger CD signals and, in many cases, a red shift in the plasmon resonance. Furthermore, left- and right-handed nanostructures exhibit mirror-image CD spectra, reflecting their opposite chiral configurations.

The optical behavior of chiral plasmonic nanoparticles can be precisely tailored by controlling their composition, size, morphology, and surface chemistry. Considerable progress has been achieved in synthesizing AuNPs with well-defined geometries, including nanospheres, nanorods, nanocubes, nanoprisms, nanotriangles, nanodiscs, octahedra, decahedra, and bipyramids. In parallel, a wide variety of chiral ligands, including amino acids [63,64], peptides [65], glutathione [63], and nucleic acids [66,67], has been employed to direct asymmetric nanoparticle growth or to transfer molecular chirality to plasmonic surfaces (Table 1). These synthetic approaches enable precise control over chiroptical activity across the visible and near-infrared spectral regions.

To establish a clearer relationship between the origin of chirality, structural characteristics, and fabrication strategies, chiral plasmonic nanomaterials can be broadly considered across four interconnected levels: surface/atomic-level chirality, intrinsic geometric chirality, hierarchical assembly induced chirality, and externally induced or dynamically regulated chirality [10,75]. Surface/atomic-level chirality originates from asymmetric ligand–metal interactions, chiral surface coordination, or stereochemical arrangements at the metal interface and can be introduced through chiral ligand functionalization or ligand-directed growth [63,76]. Intrinsic geometric chirality arises directly from the three-dimensional morphology of the plasmonic nanostructure, including twisted, helicoidal, spiral, or other enantiomorphic architectures, and is commonly generated through seed-mediated asymmetric growth, facet-selective growth, and related morphology-directed synthetic approaches [27,77]. In contrast, hierarchical assembly induced chirality originates from the asymmetric spatial organization of plasmonic building blocks into higher-order structures, where interparticle electromagnetic coupling generates or amplifies the chiroptical response [62]. Such architectures can be constructed through DNA-, peptide-, polymer-, biomolecule-, or inorganic-template-assisted assembly and through the organization of chiral nanoparticles into nanofilms and superstructures [47]. A further category involves externally induced or dynamically regulated chirality, in which external stimuli such as circularly polarized light or magnetic fields generate, enhance, or modulate chiral optical responses [9,23]. Galvanic replacement provides an additional structural transformation strategy capable of transferring or preserving pre-existing chirality while simultaneously tuning composition, porosity, and plasmonic properties.

These categories are not mutually exclusive, as a single synthetic process may involve more than one mechanism of chiral induction. For example, chiral ligands can simultaneously induce asymmetric surface interactions and direct the formation of intrinsically chiral high-index facets during seed-mediated growth. Therefore, rather than treating chirality origin and synthetic methodology as independent classifications, the following sections discuss the major fabrication strategies while emphasizing the corresponding chirality origin, structural manifestation, and resulting chiroptical characteristics. This integrated framework provides a clearer basis for understanding how synthetic control across different length scales governs the optical and functional properties of chiral plasmonic nanomaterials.

### 4.1. Structure–Chiroptical Property Relationships

The chiroptical response of plasmonic nanomaterials is strongly governed by their structural characteristics across multiple length scales. At the individual nanoparticle level, morphology and three-dimensional geometric asymmetry determine the interaction of the plasmonic modes with left- and right-handed circularly polarized light [47,75]. Twisted, helicoidal, spiral, and other intrinsically asymmetric morphologies generally produce stronger plasmonic CD than highly symmetric structures because their spatially asymmetric charge distributions support the differential excitation of plasmon modes [47]. Structural parameters such as the degree of twisting, facet orientation, groove depth, nanoscale gaps, and overall particle dimensions can therefore influence both the magnitude and spectral position of the CD response [77,78]. Increasing structural asymmetry and plasmonic confinement can enhance optical dissymmetry, whereas changes in particle size and morphology can shift the localized surface plasmon resonance and consequently alter the wavelength range of the chiroptical response.

Beyond individual nanoparticles, interparticle distance, relative orientation, and plasmonic coupling are critical determinants of chiroptical activity in assembled systems. When plasmonic nanoparticles are positioned in asymmetric configurations, near-field electromagnetic interactions between neighboring particles generate coupled plasmon modes that can substantially amplify CD signals [79]. The strength of this response is highly sensitive to nanoscale separation: decreasing the interparticle distance generally strengthens electromagnetic coupling, whereas larger separations weaken collective chiroptical effects. Relative particle orientation is equally important, as crossed, twisted, or helically displaced nanoparticles can generate strong bisignate CD responses whose sign and magnitude depend on the handedness and geometry of the assembly [80].

At larger length scales, hierarchical organization provides an additional mechanism for amplifying and tuning optical chirality. DNA origami, biomolecular templates, helical scaffolds, and ordered nanofilms can organize plasmonic building blocks into well-defined three-dimensional chiral architectures. Such hierarchical structures combine the optical responses of individual nanoparticles with long-range collective plasmonic coupling, often resulting in substantially enhanced CD and optical dissymmetry [81,82]. The number of particles, periodicity, interparticle spacing, orientation, and overall handedness of the superstructure collectively determine the resulting chiroptical response. Consequently, plasmonic chirality should be considered a multiscale phenomenon in which local morphology, interparticle electromagnetic coupling, and hierarchical structural organization act cooperatively to determine CD intensity, spectral position, and dissymmetry factor (g). Understanding these structure–property relationships is therefore essential for the rational design of chiral plasmonic systems with application-specific optical characteristics.

### 4.2. Surface- and Ligand-Induced Chirality

The development of ligand-directed chiral metal nanoclusters has provided an important strategy for generating optical activity in otherwise achiral metallic systems. Early studies demonstrated that coating gold nanoclusters with optically active glutathione (GSH) could successfully transfer molecular chirality to the inorganic framework, establishing a foundation for the design of chiral noble-metal nanomaterials [83]. Since then, this approach has been widely extended to a variety of metal nanoclusters, including gold, silver, copper, and palladium, using enantiomerically pure surface ligands. Naturally occurring and synthetic chiral molecules, such as cysteine [63,64], glutathione [84,85], penicillamine [86], N-acetylcysteine [87,88,89], and other tailor-made ligands [90,91], have all been employed to regulate the chiroptical properties of these nanostructures.

**Figure 3:**
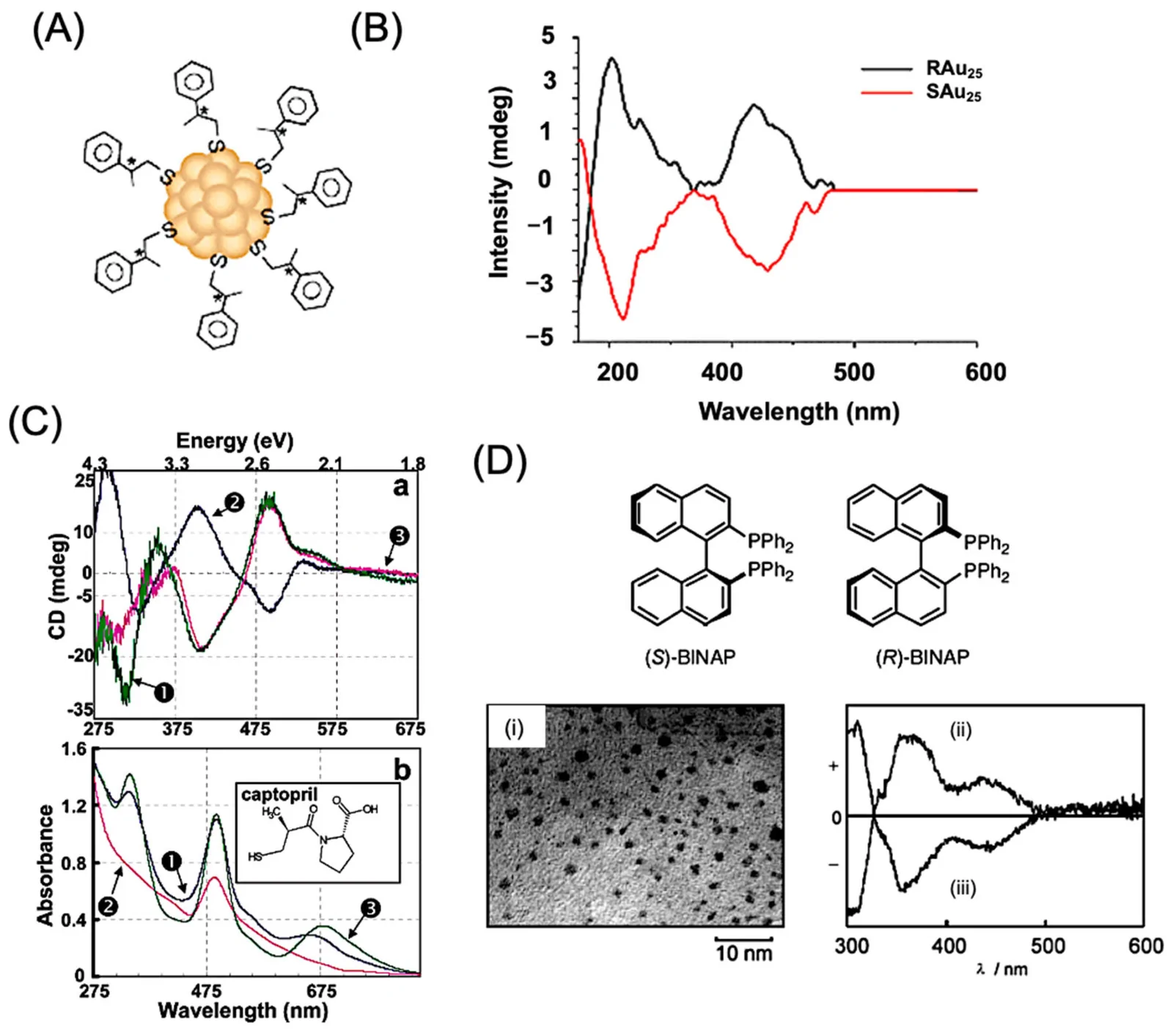
Representative examples of ligand-induced chiral noble metal nanoclusters. (**A**) Molecular structure of Au_25_(PET)_18_* nanoclusters (PET* = SCH_2_C*H(Me)Ph). * Indicate the chiral center. (**B**) CD spectra of the R- and S-Au_25_ enantiomers showing mirror-image chiroptical responses. (**C**) CD and UV–Vis spectra of silver nanoclusters stabilized with (1) captopril, (2) glutathione, or (3) mixed captopril/glutathione ligands (62:38); inset shows captopril structure. (**D**) BINAP-functionalized Au nanoparticles: BINAP molecular structure, (**i**) TEM images, and CD spectra of (**ii**) (R)-BINAP-Au and (**iii**) (S)-BINAP-Au in chloroform, demonstrating opposite optical activity. Panel (**A**,**B**) reproduced with permission from [92]; Copyright 2011, American Chemical Society. Panel (**C**) reproduced with permission from [93]; Copyright 2010, American Chemical society. Panel (**D**) reproduced with permission from [94]; Copyright 2003, American Chemical Society.

The origin of chirality in ligand-protected nanoclusters is generally attributed to strong electronic interactions at the metal–ligand interface. Upon binding to the nanoparticle surface, chiral ligands create an asymmetric local electronic environment that perturbs the electronic states of the otherwise symmetric metal core, resulting in characteristic CD responses. A representative example was reported by Zhu and co-workers, who synthesized atomically precise Au_525_(SR)_18_ nanoclusters using both R- and S-configured thiol ligands (Figure 3A) [92]. The resulting enantiomeric nanoclusters exhibited mirror-image CD spectra, demonstrating that the observed optical activity originated from electronic coupling between the surface-bound ligands and the outer gold atoms rather than from the metallic core alone (Figure 3B). This ligand-mediated chiral induction is commonly interpreted using the dissymmetric field model, in which the asymmetric electrostatic field generated by the adsorbed chiral molecules perturbs the electronic structure of the metal nanocluster and removes its intrinsic symmetry, thereby producing measurable chiroptical activity. Importantly, the magnitude and even the sign of the CD response are highly dependent on ligand identity, surface binding geometry, and ligand packing. Consequently, modifying the surface chemistry offers a versatile means of tuning nanoparticle chirality. For example, Cathcart and colleagues demonstrated that incorporating mixtures of different chiral ligands on silver nanoclusters enabled controllable modulation of their chiroptical properties, illustrating that ligand composition provides an effective handle for engineering and optimizing optical chirality (Figure 3C) [93]. The use of chiral reagents such as R/S-BINAP can induce chirality in Au and Pd NPs, which are in turn used for chiral catalytic applications such as the hydrosilylation of styrene (Figure 3D) [94]. These NPs showed excellent enantioselective hydrosilylation under mild conditions.

Recent investigations have highlighted the growing potential of chiral metallic nanoparticles in biomedical and biosensing applications. For example, Zhang and co-workers demonstrated that D- and L-cysteine-functionalized gold nanoparticles can serve as efficient nanocarriers for immunoadjuvant delivery [95]. Interestingly, nanoparticles modified with L-cysteine elicited a significantly stronger immune response than their D-cysteine counterparts, producing more than a 66-fold enhancement in anti-ovalbumin (OVA) IgG antibody levels, thereby underscoring the critical role of nanoparticle chirality in regulating immune activation. In another study, Maniappan et al. synthesized L- and D-cysteine-stabilized copper nanoparticles and investigated their ability to discriminate chiral amino acids [96]. Among the analytes examined, L-histidine exhibited a remarkable inversion of the circular dichroism signal upon interaction with the chiral copper nanoparticles, demonstrating their potential as highly sensitive platforms for enantioselective molecular recognition. Chiral palladium nanoparticles were also prepared using such a strategy, in which chiral amino acids such as proline were used as chiral modifiers to make dendritic or cubic palladium nanocrystals. These nanocrystals were used for asymmetric hydrogenation reactions [97]. Binaphthyl-derived chiral phosphoramidite ligands have been employed to stabilize palladium nanoparticles, producing highly enantioselective heterogeneous catalysts for asymmetric Suzuki C–C cross-coupling reactions [98]. The chiral ligand shell creates an asymmetric catalytic environment at the nanoparticle surface, enabling efficient stereo control while maintaining the high catalytic activity and recyclability associated with nanoparticle-based catalysts. This work demonstrates that ligand-engineered metal nanoparticles can combine the advantages of homogeneous chiral catalysts with the robustness of heterogeneous nanocatalysts, highlighting an effective strategy for developing enantioselective nanocatalytic systems.

Direct synthetic approaches offer a straightforward route for producing chiral gold nanoparticles because they generally involve fewer processing steps and relatively short reaction times. These methods enable the simultaneous formation of the nanoparticle core and its chiral characteristics within a single synthesis process, making them attractive for rapid and scalable preparation. Nevertheless, achieving consistent chirality requires precise regulation of reaction parameters, including precursor concentration, ligand chemistry, temperature, and growth kinetics. The complex interplay of these factors often limits reproducibility and makes it challenging to finely tune nanoparticle size, morphology, and chiroptical activity. In addition, directly synthesized chiral nanoparticles may exhibit broader size distributions and reduced structural stability, which can affect the uniformity and long-term performance of their chiral properties.

### 4.3. Chiral Nanomaterials by Seed-Mediated Method

**Figure 4:**
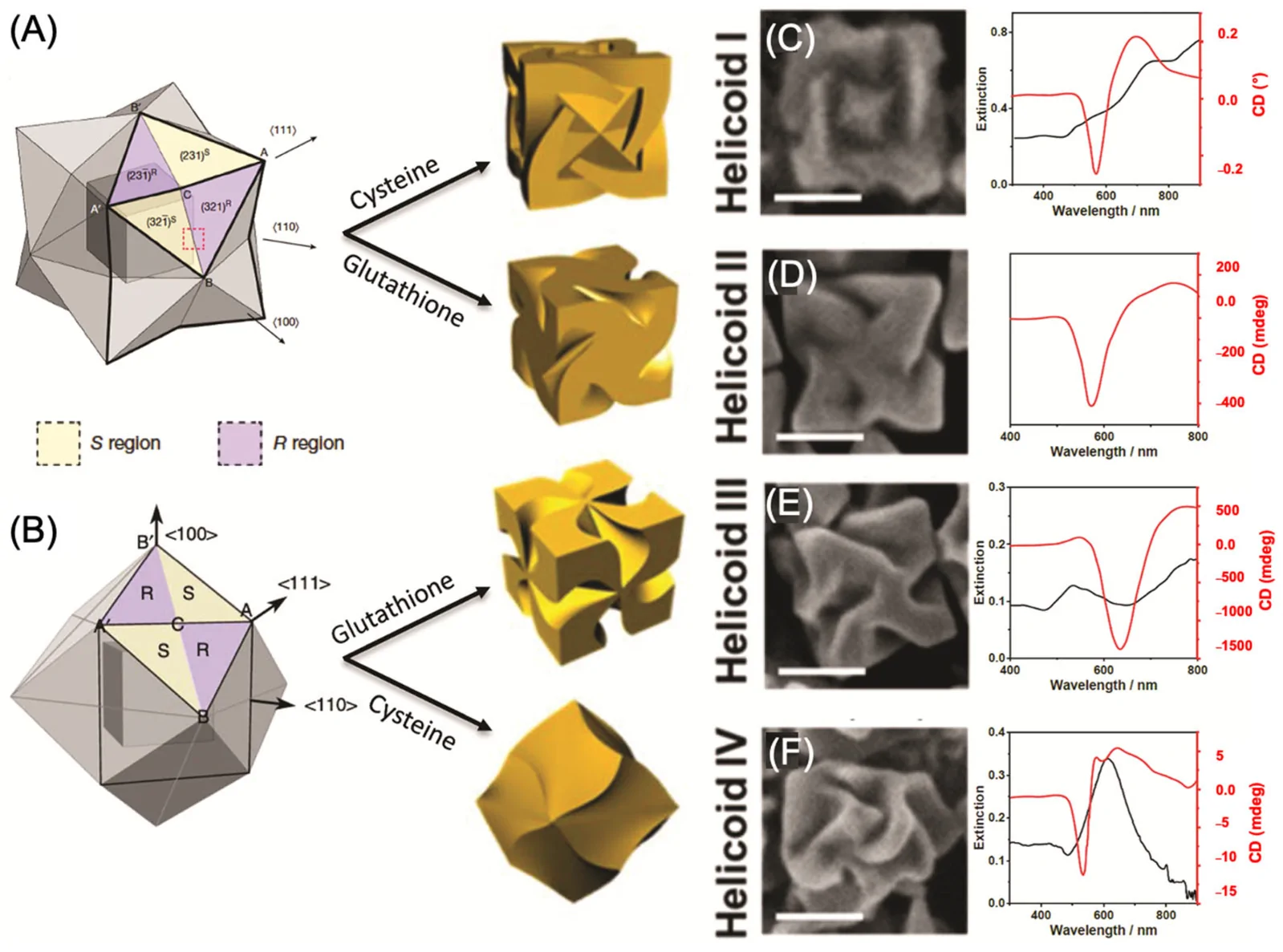
Chiral nanostructures using seed-mediated method. (**A**,**B**) Schematic illustration showing the structural evolution of stellated octahedral (**A**) and rhombic dodecahedral (**B**) gold nanocrystals containing right-handed (R) and left-handed (S) high-index surface domains. The presence of chiral ligands, including cysteine and glutathione, directs asymmetric crystal growth, resulting in distinct helicoidal plasmonic nanostructures. The representative rhombic face is defined by the symmetry-related vertex pairs A/A′ and B/B′, while C denotes the intersection of the AA′ and BB′ diagonals and hence the center of the rhombus. The prime symbol (′) identifies the symmetry-equivalent counterpart of each vertex. (**C**–**F**) Representative scanning electron microscopy (SEM) images together with the corresponding UV–Vis absorption and circular dichroism (CD) spectra of the synthesized chiral gold nanoparticles: 432 Helicoid I (**C**), 432 Helicoid II (**D**), 432 Helicoid III (**E**), and 432 Helicoid IV (**F**). Panel (**A**–**F**) reproduced with permission from [63]; Copyright 2018, Nature publishing group.

Studies have demonstrated that incorporating enantiomerically pure ligands during nanoparticle formation can effectively transfer molecular chirality to plasmonic materials, resulting in significant optical activity [99]. Among these approaches, Lee et al. introduced an amino acid- and peptide-assisted synthetic strategy in which selective adsorption of chiral biomolecules onto specific crystallographic facets guided anisotropic nanoparticle growth [63]. The differential interactions between the ligands and the evolving gold surface promoted the formation of highly twisted helicoidal nanostructures exhibiting intense plasmonic chiroptical properties. The resulting nanoparticles displayed strong CD signals, reaching approximately 0.5 mdeg, with a corresponding dissymmetry factor of nearly 0.2. In this methodology, small gold nanocrystals serve as seeds that are subsequently grown in the presence of cysteine or other chiral molecular precursors, enabling controlled development of well-defined chiral architectures. This synthetic route is commonly referred to as the seed-mediated growth strategy. The formation of these chiral plasmonic nanostructures is governed by the enantioselective interaction between chiral ligands and high-index gold crystal facets, which directs asymmetric crystal growth during a seed-mediated synthesis. Typically, cubic gold nanoparticles enclosed by low-index {100} facets are used as seeds and subsequently overgrown in the presence of chiral amino acids or peptides. Selective adsorption of ligands through thiol, amino, and carboxyl groups modifies the growth kinetics of specific crystallographic planes, resulting in twisted helicoidal nanostructures with pronounced plasmonic circular dichroism. In the absence of chiral ligands, seed overgrowth produces achiral stellated octahedra composed of alternating right- and left-handed {321} facets (Figure 4A). However, the addition of L- or D-cysteine selectively promotes the growth of one chiral domain over the other, generating 432 Helicoid I nanoparticles with twisted edges and nanoscale gaps (Figure 4B,C), exhibiting a dissymmetry factor (g) of approximately 0.03. Replacing cysteine with glutathione alters the crystal growth pathway, leading to a fourfold symmetric pinwheel-like 432 Helicoid II morphology with an improved g-factor of ~0.05 (Figure 4D). Further enhancement was achieved by employing octahedral gold seeds together with glutathione, producing 432 Helicoid III nanoparticles characterized by highly twisted pinwheel architectures and deeper radial nanogaps (Figure 4E). These nanostructures exhibited a significantly stronger chiroptical response, with g-factors reaching ~0.20, which were subsequently increased to 0.31 through optimized multistep growth and improved morphological uniformity. The use of cysteine with octahedral seeds resulted in a Helicoid-IV-type structure (Figure 4F). Extending this Kumar et al. showed the use of various amino acids for the generation of various Helicoid structures and used them for enantioselective recognition of chiral analytes using Surface-Enhanced Raman Spectroscopy (SERS) [64].

Despite significant advances in nanoparticle synthesis, the controlled preparation of intrinsically chiral silver (Ag) nanoparticles remains considerably more challenging than that of their gold counterparts [100]. One of the primary obstacles is the formation of chiral high-index crystal facets, characterized by Miller indices where h, k, and l are all different and nonzero. These crystallographic planes are widely regarded as essential structural motifs for generating nanoscale chirality because they provide the asymmetric atomic arrangements required for helical or enantiomorphic surface architectures. However, such facets are rarely observed in silver nanocrystals due to the high surface reactivity of Ag and the limited availability of chiral ligands capable of selectively stabilizing these energetically unfavorable surfaces during crystal growth.

To address these limitations, researchers have explored the use of chiral amino acids and biomolecular ligands, particularly L- and D-cysteine, as structure-directing agents [101,102]. Since the nucleation of silver typically requires rapid reduction, whereas controlled growth of chiral facets favors slower kinetic conditions, seed-mediated growth has become the preferred synthetic strategy. In this approach, preformed metallic seeds—most commonly gold nanoparticles—serve as crystallographic templates that enable the gradual epitaxial deposition of silver under milder reaction conditions. Beyond facilitating controlled shell growth, the use of Au seeds also provides a convenient platform for monitoring the evolution of silver deposition and understanding the underlying growth mechanism.

**Figure 5:**
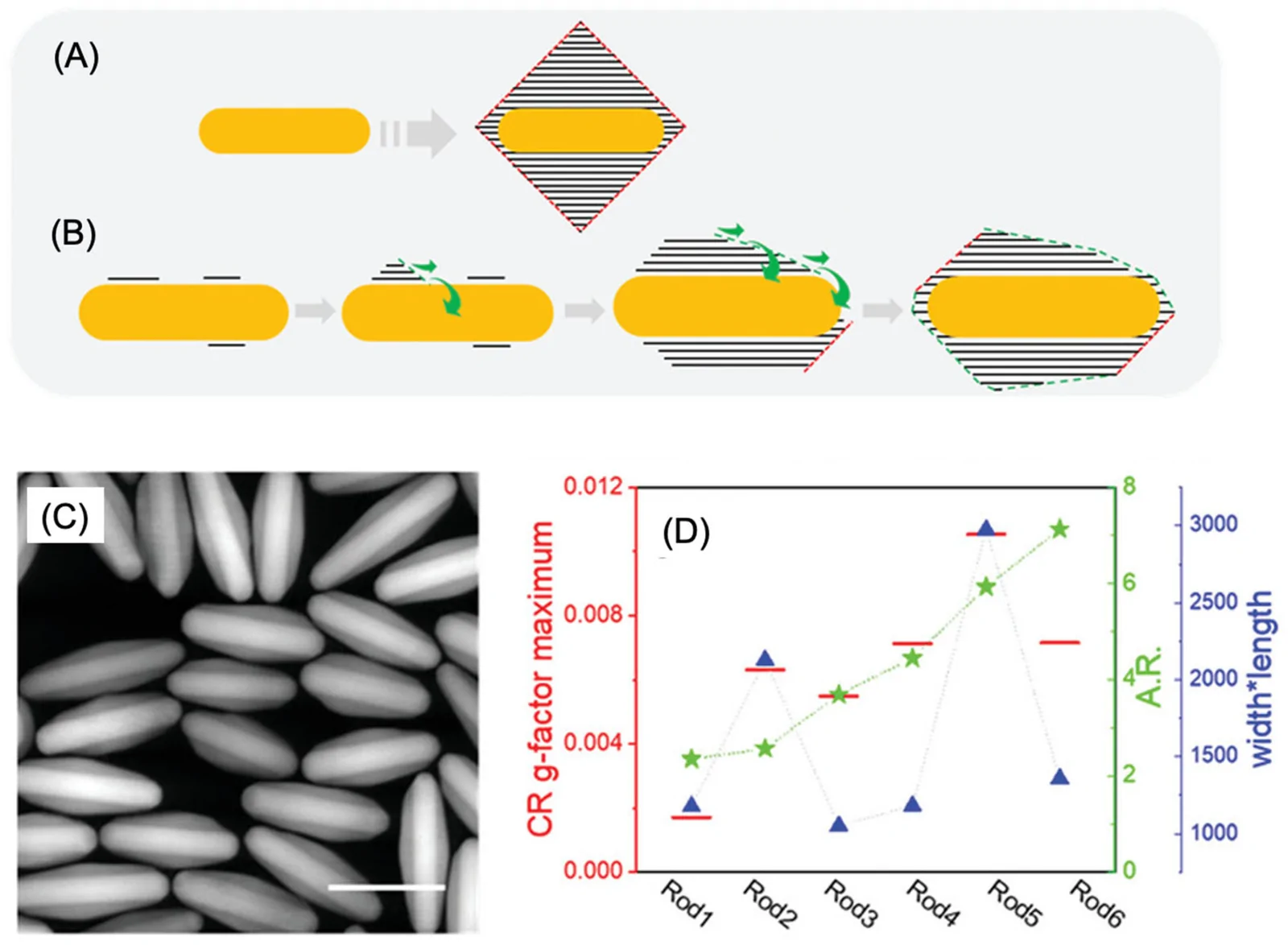
Growth mechanism and chiroptical characteristics of seed-mediated Au@Ag chiral nanorods. (**A**) Schematic evolution of Au@Ag nanostructures into truncated octahedral morphologies during Ag shell growth. (**B**) Proposed Ag growth pathway showing newly deposited Ag layers (black), {111} facets (red dashed lines), surface irregularities (green dashed lines), and growth fronts (green arrows). (**C**) STEM image of chiral rod 5 (CR5) (scale bar: 100 nm). (**D**) Correlation between maximum chiroptical g-factor and Au nanorod seed dimensions, illustrating the effect of seed geometry on optical dissymmetry. Reproduced with permission from [103]; Copyright 2023, Wiley Publishers.

Several studies have demonstrated that cysteine-assisted seed-mediated synthesis can generate Au@Ag core–shell nanostructures exhibiting transient chiral morphologies. A representative example involves the formation of truncated octahedral Au@Ag nanoparticles, where detailed mechanistic investigations revealed that chirality emerges during intermediate growth stages rather than in the final equilibrium morphology [103]. Initially, reduced Ag atoms nucleate heterogeneously on the seed surface, producing irregular protrusions and anisotropic surface features (Figure 5A). As silver deposition proceeds and the precursor concentration gradually decreases, particle growth slows and ultimately terminates when the available Ag ions are exhausted (Figure 5B,C). During this evolution, the selective adsorption of cysteine onto specific crystallographic planes alters the relative growth rates of different facets, temporarily stabilizing asymmetric truncated geometries before the system evolves toward more thermodynamically favored structures (Figure 5C). Although the fully developed particles often approach highly symmetric octahedral morphologies, these intermediate truncated nanostructures retain distinct chiral characteristics arising from enantioselective ligand–surface interactions. The mechanistic insights obtained from Au@Ag systems also suggest that similar growth pathways could be realized using silver seeds, enabling the direct synthesis of entirely silver-based chiral nanoparticles without a gold core. Furthermore, the degree of structural asymmetry appears to depend strongly on the geometric characteristics of the initial seed. Seeds possessing larger surface areas provide more nucleation sites and allow localized growth anisotropy to persist under a finite precursor supply, while elongated seeds with higher aspect ratios inherently promote asymmetric deposition. Consequently, the magnitude of nanoparticle dissymmetry is expected to increase with both the available seed surface area and the seed aspect ratio. Experimental observations have consistently supported this relationship, demonstrating that careful control of seed geometry provides an effective strategy for tuning the chirality and optical activity of silver-based nanostructures (Figure 5D).

Seed-mediated synthesis is a powerful strategy for producing chiral nanomaterials with precise control over size, morphology, and surface structure. By separating nucleation from growth, it enables the formation of well-defined asymmetric nanostructures with tunable optical and catalytic properties. However, the method remains highly sensitive to reaction conditions, seed quality, and ligand chemistry, making reproducibility and large-scale production challenging. Future efforts should focus on improving synthetic robustness, scalability, and enantiomeric control to accelerate practical applications.

### 4.4. Chiral Nanomaterials by Template-Assisted Synthesis or Assemblies

Chiral soft templates, including self-assembled micelles [68], DNA [82], and biopolymer scaffolds [104], provide an effective platform for directing the growth of well-defined helical and superlattice nanostructures. Their structural organization enables the fabrication of plasmonic nanomaterials with strong collective chiroptical responses, making them attractive for applications in surface-enhanced Raman scattering (SERS), optical metamaterials, and chiral bioimaging. A notable example is the work by Liz-Marzán and co-workers, who employed chiral surfactant-derived micelles as growth-directing templates for seed-mediated synthesis of anisotropic gold nanostructures [68]. The helical micellar assemblies guided asymmetric metal deposition, producing grooved chiral morphologies with exceptionally strong circular dichroism, reaching g-factors of approximately 0.2 in the near-infrared region. The pronounced optical activity originated from the twisted plasmonic architecture of the nanoparticles. Furthermore, modulation of the concentration of chiral ligands and other growth parameters during seed-mediated synthesis enabled control over, and even reversal of, the plasmonic chiral response [73].

**Figure 6:**
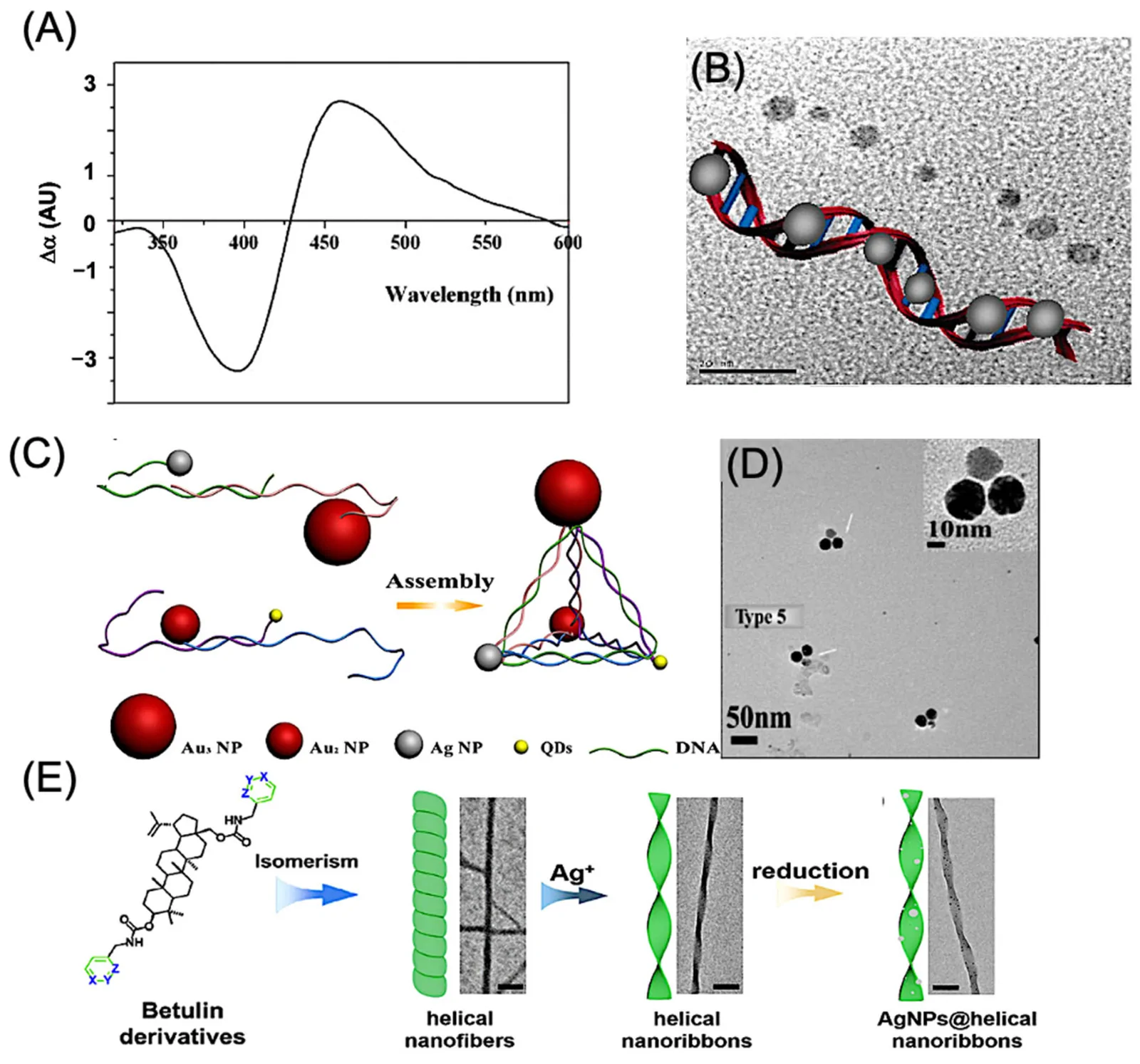
DNA- and biomolecule-assisted fabrication of chiral silver nanostructures. (**A**) CD spectra of Ag nanoparticles synthesized on DNA templates. (**B**) TEM image showing DNA-directed linear assembly of Ag nanocrystals. (**C**) DNA-guided assembly of chiral nanoparticle pyramids and representative TEM image. (**D**) TEM images of self-assembled pyramidal structures composed of S-Au_2_, Au_3_, Ag, and quantum dots (QDs). (**E**) Biomolecule-templated synthesis of AgNP-decorated helical nanoribbons. Panels (**A**,**B**) reproduced with permission from [106]; Copyright 2006, American Chemical Society. Panels (**C**,**D**) reproduced with permission from [107]; Copyright 2012, American Chemical Society. Panel (**E**) reproduced with permission from [108]; Copyright 2022, American Chemical Society.

Biological molecules have emerged as versatile templates for the fabrication of chiral nanomaterials because of their intrinsic chirality, biocompatibility, and ability to direct nanoparticle assembly under mild conditions. Among these, DNA has emerged as one of the most versatile scaffolds because of its predictable helical structure and programmable molecular recognition [105]. While DNA-directed synthesis of chiral Au nanoparticles was first demonstrated in the late 1990s, the preparation of DNA-templated chiral Ag nanoparticles was achieved several years later owing to the greater synthetic complexity associated with silver. In a typical approach, Ag^+^ ions are coordinated along double-stranded DNA and subsequently reduced to generate ~5 nm Ag nanoparticles uniformly distributed along the DNA helix (Figure 6A) [106]. The periodic arrangement of Ag nanoparticles within the helical framework promotes strong plasmonic coupling, producing characteristic bisignate circular dichroism signals in the visible region (Figure 6B). Beyond serving as a structural template, DNA also functions as a programmable assembly ligand that enables the hierarchical organization of Ag nanoparticles into complex three-dimensional chiral architectures. DNA-functionalized nanoparticles can undergo sequence-specific hybridization and thermal annealing to form ordered superstructures, including chiral pyramids and other enantiomeric assemblies with precisely controlled handedness (Figure 6C,D) [107]. This strategy provides exceptional spatial precision and enhanced chiroptical activity, with anisotropy (*g*) factors approaching 10^−2^**,** significantly exceeding those typically observed for small chiral molecules. Despite these advantages, DNA-directed assembly remains limited by the high cost of synthetic DNA, sensitivity to temperature and sample purity, and the need for carefully controlled experimental conditions. Besides DNA, naturally derived chiral biomolecules have also been employed as templates for silver nanostructure growth. Chiral triterpenoid-based ligands can self-assemble with Ag^+^ ions into helical supramolecular frameworks that serve as templates for nanoparticle formation (Figure 6E) [108]. Upon reduction, Ag nanoparticles nucleate along these helical scaffolds while preserving the underlying chiral organization, producing stable plasmonic nanostructures with pronounced CD responses. This biomolecule-assisted strategy provides a simple and mild synthetic route with good structural control; however, precise optimization of ligand concentration, metal-ion ratio, and irradiation conditions is essential to achieve reproducible chirality and optical performance.

**Figure 7:**
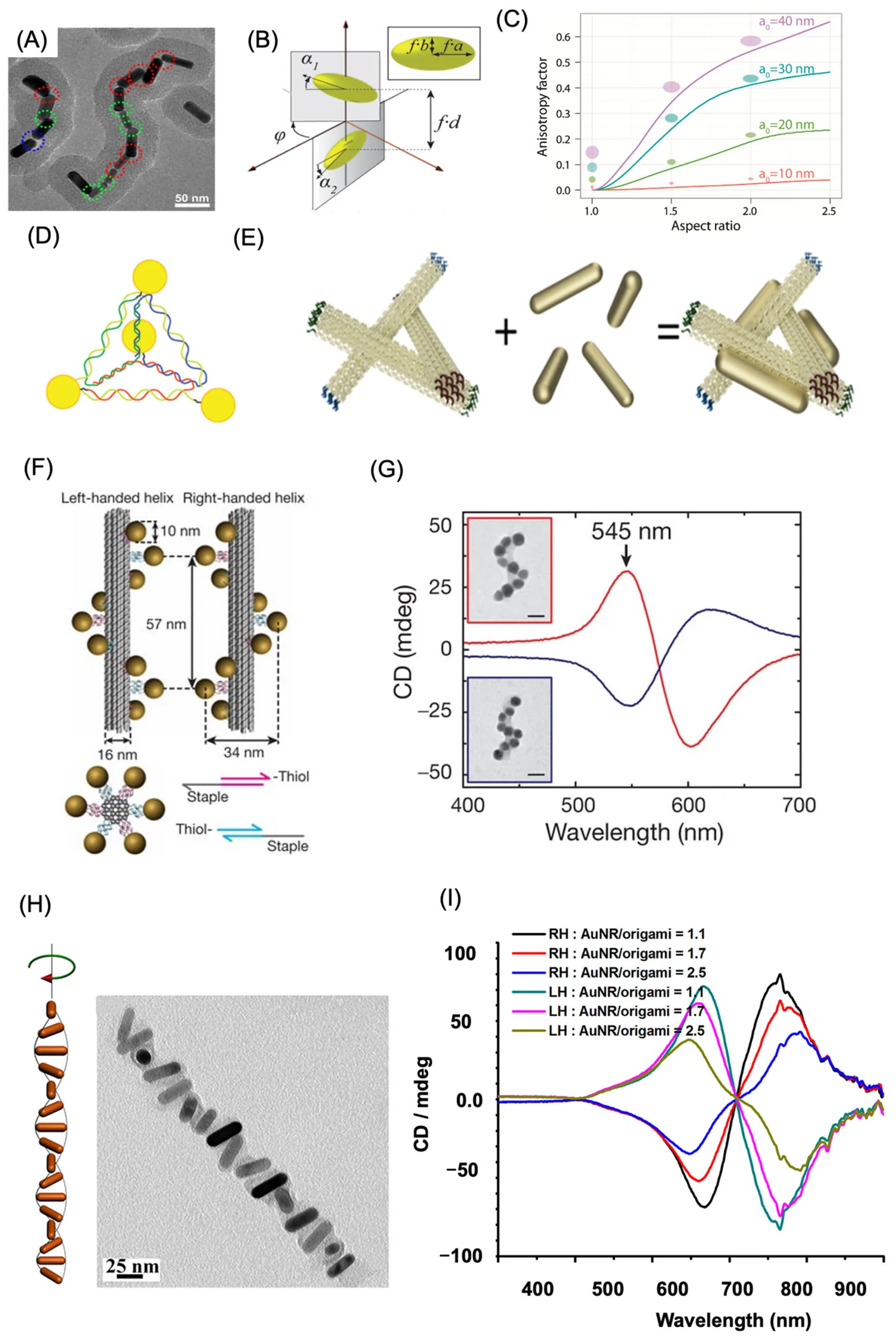
Template-assisted assembly strategies for chiral plasmonic nanostructures. (**A**) TEM image of an end-to-end crossed (EEX) gold nanostructure. The Nanorod junctions corresponding to a left-handed edge-to-edge crossed (LH-EEX) junction, a right-handed edge-to-edge crossed (RH-EEX) junction, and a parallel edge-to-edge junction (PEEJ) are indicated by green, red, and blue circles, respectively. (**B**) Schematic of an AuNR dimer. (**C**) Coupled-dipole simulations of the anisotropy factor (g) as a function of AuNR size and aspect ratio. (**D**) DNA nanocrystal pyramid for chiral plasmonic assembly. Yellow spheres indicate respective gold nanoparticles at the edge of a pyramidal structure. (**E**) AuNR assembly on DNA origami. (**F**) Left- and right-handed DNA origami-templated AuNR nanohelices. (**G**) Enhanced CD arising from collective plasmonic coupling. (**H**) Structural models and TEM images and (**I**) CD spectra of right- and left-handed AuNR helices with varying AuNR/origami ratios. Panel (**A**) reproduced with permission from [110]; Copyright 2017, American Chemical Society. Panel (**B**,**C**) reproduced with permission from [111]; Copyright 2011, American Chemical Society. Panel (**D**), reproduced with permission from [112]; Copyright 2009, American Chemical Society. Panel (**E**) reproduced with permission from [81]; Copyright 2025, American Chemical Society. Panel (**F**,**G**) reproduced with permission from [115]; Copyright 2013, Nature Publishing group. Panel (**H**,**I**) is reproduced from [116]; Copyright 2015, American Chemical Society.

Template-directed assembly has emerged as an effective strategy for constructing plasmonic nanostructures with well-defined chirality [109]. Liu et al. demonstrated that chiral templates can direct the formation of end-to-end crossed (EEX) nanoparticle assemblies, producing pronounced CD responses (Figure 7A) [110]. Likewise, Liz-Marzán and co-workers fabricated crossed dimers of gold nanorods (Figure 7B,C), where the asymmetric CD spectra originated from plasmonic coupling between the closely positioned nanorods [111]. Computational simulations closely matched the experimental CD profiles, confirming the role of electromagnetic interactions in generating the observed optical activity. DNA has become one of the most versatile scaffolding materials for organizing plasmonic nanoparticles with nanometer-scale precision. By exploiting the inherent tetrahedral symmetry of DNA frameworks, researchers assembled pyramidal nanoparticle architectures in which the spatial arrangement and orientation of nanocrystals could be precisely controlled, leading to well-defined chiral geometries (Figure 7D) [112]. Beyond structural organization, DNA-mediated assembly has also been extended to biosensing applications. For example, Xu et al. employed antibody–antigen interactions to bridge silver and gold nanoparticles into chiral AgNP–AuNP dimers, enabling sensitive detection of environmental contaminants such as microcystin-LR as well as cancer-associated biomarkers [113].

Recent advances in DNA origami have further expanded the ability to engineer sophisticated chiral plasmonic systems by enabling programmable placement of nanoparticles with exceptional spatial accuracy [114]. Using a left-handed DNA origami template composed of three interconnected 14-helix bundles (14HBs), gold nanorods were assembled into chiral architectures through hybridization between A19-functionalized DNA strands and poly-T-modified nanorods (Figure 7E) [81]. Connector oligonucleotides linked individual origami units into larger plasmonic assemblies with controlled handedness. Building on this concept, Kuzyk and co-workers employed DNA origami to fold long single-stranded DNA scaffolds into predefined two- and three-dimensional architectures that served as templates for helical gold nanoparticle assemblies (Figure 7F) [115]. These structures exhibited strong circular dichroism and optical rotatory dispersion throughout the visible spectrum arising from collective plasmonic interactions among the helically arranged nanoparticles (Figure 7G). The helical geometry promotes directional propagation of coupled plasmon modes, resulting in preferential interaction with circularly polarized light that matches the handedness of the assembly. Importantly, theoretical calculations accurately reproduced both the spectral positions and intensities observed experimentally, confirming the structural origin of the chiroptical response. Wang et al. further demonstrated programmable assembly of anisotropic helical superstructures by positioning gold nanorods on two-dimensional DNA origami templates decorated with strategically arranged 15-nucleotide capture strands in an X-shaped configuration [116]. The resulting nanorod helices displayed exceptionally strong chiroptical activity, with anisotropy factors reaching approximately 0.02 (Figure 7H,I). Beyond helical architectures, DNA origami has also enabled the fabrication of numerous other chiral plasmonic nanostructures, including gold nanoparticle tetramers [117], bipyramidal assemblies [118], gold nanorod dimers [119], and a variety of other programmable nanoscale configurations.

Beyond molecular templates, inorganic chiral templates provide an effective route for constructing plasmonic nanostructures with well-defined three-dimensional architectures. For example, Correa-Duarte and co-workers developed chiral silica nanoribbons as scaffolds for assembling TiO_2_ and Au nanoparticles into hybrid photocatalytic systems [120]. Following amine functionalization, spherical Au nanoparticles were covalently anchored onto the twisted silica surface, generating chiral plasmonic assemblies. The helical arrangement of Au nanoparticles promoted strong plasmonic coupling and polarization-dependent photocatalytic activity under circularly polarized light, where hot-carrier generation was governed by the light helicity. Cheng et al. fabricated three-dimensional helical plasmonic nanostructures by assembling AuNPs on silica nanohelices [121]. The chiroptical response was strongly influenced by both the size and spatial arrangement of the AuNPs, yielding an anisotropy factor (g-factor) of ~1 × 10^−4^ at the SPR wavelength, approximately ten times higher than previously reported values. Another widely adopted strategy is in situ nucleation and growth, where metal precursors are adsorbed onto preformed chiral templates and subsequently reduced to generate chiral nanostructures. Using this approach, Che et al. synthesized chiral mesoporous silica templates that directed the growth of Ag nanoparticles with tunable CD responses, producing Ag–silica composites exhibiting strong circular dichroism in the visible region [122].

Template-assisted strategies offer exceptional control over chiral nanoparticle assembly and reproducible optical properties but are often limited by complex fabrication procedures, poor scalability, and high production costs.

### 4.5. Chiral Nanomaterials by Nanofilms

**Figure 8:**
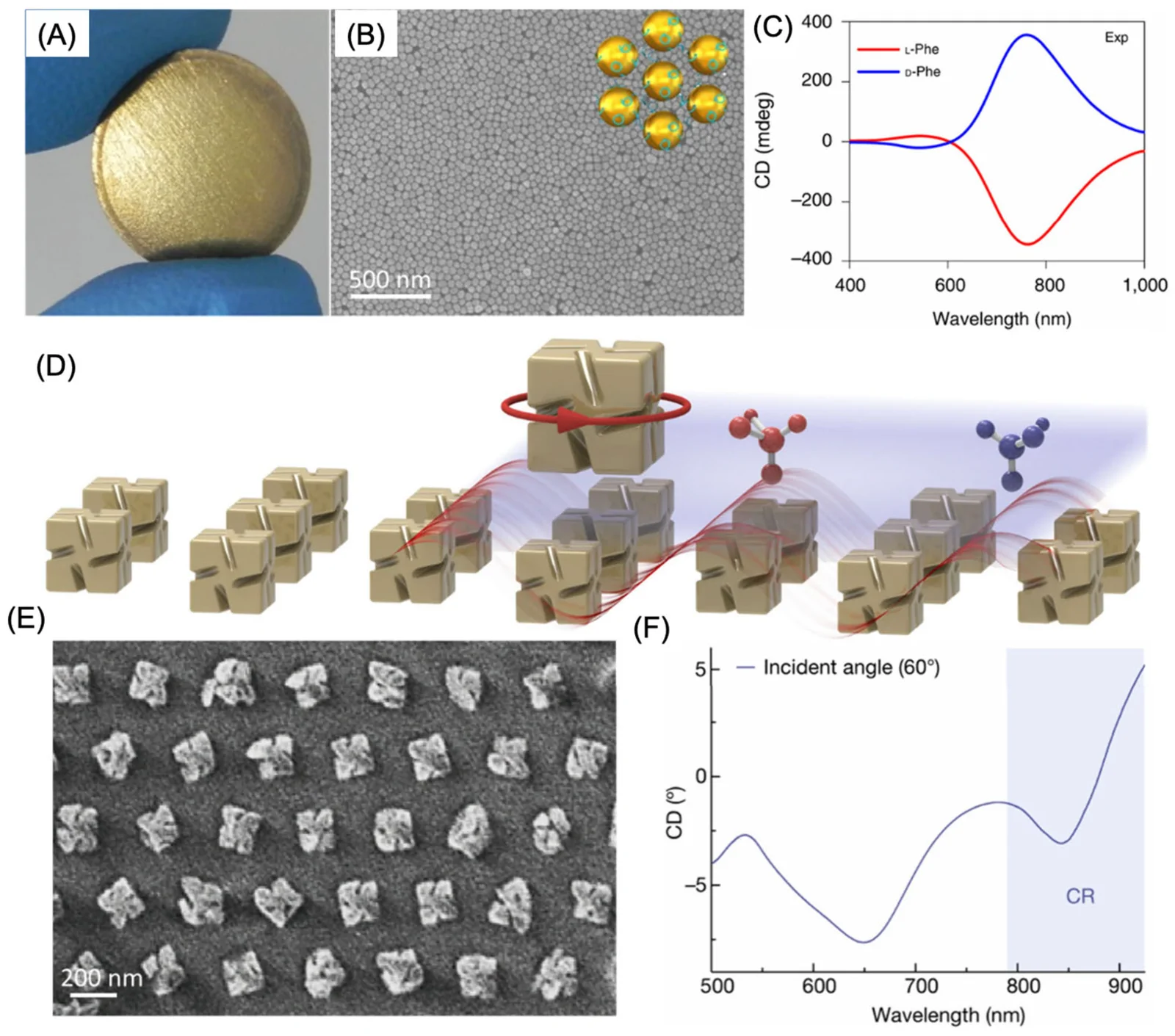
Bottom-up fabrication of chiral plasmonic nanofilms. (**A**) Photograph of a macroscopic chiral nanofilm. (**B**) SEM image of the left-handed (L-type) nanofilm. (**C**) Circular dichroism (CD) spectra demonstrating the enhanced chiroptical response of the assembled nanofilm. (**D**) Schematic illustration of a two-dimensional (2D) helicoid crystal assembled from chiral Au nanoparticles. (**E**) SEM image showing the hexagonal arrangement of helicoids into a periodic 2D crystal. (**F**) CD spectra illustrating the enhancement of collective plasmonic resonances in the 2D helicoid assembly. Panel (**A**–**C**) reproduced with permission from [128]; Copyright 2022, Nature Publishers. Panel (**D**–**F**) reproduced with permission from [124]; Copyright 2022, Nature Publishers.

Compared with individual chiral plasmonic nanoparticles, chiral nanofilms exhibit enhanced collective optical responses due to long-range nanoparticle ordering, leading to stronger CD and improved light–matter interactions [11]. These unique properties make them attractive for optical sensing, polarization control, and photonic devices. Fabrication strategies are generally classified into bottom-up and top-down approaches. Bottom-up assembly relies on solution-based methods such as interfacial self-assembly and the Langmuir–Schaefer technique, enabling scalable fabrication of ordered chiral films. For example, Xu et al. assembled L/D-phenylalanine-functionalized Au nanoparticles into chiral monolayers at a liquid–liquid interface using the Langmuir–Schaefer method (Figure 8A,B) [123]. The resulting nanofilms exhibited significantly enhanced CD signals compared with dispersed nanoparticles, demonstrating that collective nanoparticle organization amplifies chiroptical activity (Figure 8C). Similarly, Nam and co-workers employed helicoidal Au nanoparticles as building blocks to fabricate highly ordered two-dimensional helical crystals through dip-coating and directed assembly (Figure 8D–F) [124]. The resulting superstructures showed approximately 2.5-fold enhancement in CD intensity due to collective plasmonic coupling and were further extended into multilayer chiral Au nanofilms. Top-down strategies provide superior structural precision through techniques such as in situ growth [125], electron-beam lithography [126], and holographic lithography [127]. Che and co-workers synthesized chiral Au, Ag, and Cu nanofilms by directing the helical assembly of metal nanoparticles using N-acetyl-L/D-cysteine, producing inorganic chiral films with morphology-dependent plasmonic and scattering-induced optical activity [128]. In another study, Kadodwala and co-workers fabricated periodic fylfot-shaped Au nanostructures using electron-beam lithography [126]. These intrinsically chiral nanostructures exhibited strong CD responses arising from structural asymmetry and localized surface plasmon resonance, although practical implementation is limited by fabrication complexity and high production costs. Overall, bottom-up approaches offer scalable and cost-effective fabrication of large-area chiral nanofilms, whereas top-down techniques provide exceptional structural precision and reproducibility. Nevertheless, improving scalability while maintaining precise chiral architectures remains a key challenge for translating chiral nanofilms into practical optical and biomedical applications.

### 4.6. Circularly Polarized Light (CPL)-Induced Chiral Growth and Chirality Switching

Circularly polarized light (CPL) has emerged as a powerful external chiral stimulus for directing the synthesis and dynamic regulation of plasmonic nanostructures. Unlike conventional molecular templating approaches, CPL transfers optical chirality directly to metallic nanomaterials, enabling asymmetric nucleation, anisotropic crystal growth, and controlled chiroptical responses. Early studies by Kotov and co-workers demonstrated that irradiation of Au^3+^ precursor solutions with CPL produced gold nanoparticles that spontaneously assembled into chiral superstructures [129]. Subsequently, Xu and co-workers employed CPL-assisted seed-mediated growth to fabricate chiral Au nanoparticles with exceptionally high optical anisotropy factors (g ≈ 0.4), highlighting the ability of polarized light to precisely control nanoscale chirality [130]. Beyond directing nanoparticle growth, CPL also enables site-selective morphological evolution through localized plasmonic excitation. Lee and co-workers showed that the combination of CPL and chiral amino acids selectively modulates active growth sites on Au nanoparticles, resulting in enhanced or suppressed circular dichroism (CD) depending on the handedness of the incident light [131]. Moreover, alternating between left- and right-handed CPL allows reversible chirality switching, accompanied by inversion of the corresponding optical signals, demonstrating dynamic control over chiral plasmonic systems. Recent advances have further expanded CPL-mediated synthesis to hybrid nanostructures. Tatsuma and co-workers exploited CPL-generated plasmonic hot carriers to achieve asymmetric deposition of PbO_2_ on Au nanorods supported on TiO_2_, producing mirror-image chiral architectures through handedness-dependent localization of charge carriers [132,133]. Complementary theoretical studies by Govorov and co-workers revealed that the spatial distribution of plasmon-induced hot electrons governs non-uniform crystal growth under CPL, providing a mechanistic framework for light-driven chirality generation [132]. More recently, the synergistic combination of CPL irradiation with chiral molecular ligands has enabled the fabrication of plasmonic nanostructures with even stronger chiroptical responses (g values up to 0.44), offering a promising strategy for designing multifunctional chiral hybrid nanomaterials with precisely tunable optical properties [130].

### 4.7. Magnetic-Field-Induced Magneto-Optical and Chiroptical Responses

It is important to distinguish geometric chirality from magnetic-field-induced optical activity. Geometrically chiral nanostructures possess intrinsic handedness arising from their asymmetric three-dimensional morphology or spatial organization and can therefore exhibit chiroptical responses even in the absence of an external magnetic field. In contrast, an applied magnetic field can break time-reversal symmetry and produce non-reciprocal magneto-optical responses, including differential interactions with circularly polarized light, even in nanostructures that are not geometrically chiral. Such effects should therefore not be interpreted as the generation of structural chirality by the magnetic field. The integration of magnetic components with optically active nanomaterials provides an attractive strategy for externally controlling these magneto-optical and chiroptical responses. However, the development of discrete hybrid nanoparticles remains challenging because magnetic materials generally interact weakly with incident light and because integration with semiconductor or plasmonic components can introduce substantial interfacial and lattice mismatch. One promising approach involves the regioselective incorporation of magnetic domains into semiconductor nanostructures, although lattice mismatch between magnetic and semiconducting materials presents a significant synthetic challenge [134]. A notable example was reported by Yu, Tang, Sargent, and co-workers, who fabricated hybrid semiconductor nanorods with Fe_3_O_4_ magnetic nanodomains selectively grown at one end through an Au/Ag_2_S buffer-layer strategy [135]. The intermediate layers reduced interfacial incompatibility, enabling controlled assembly of the multicomponent heterostructure. Importantly, these hybrid nanostructures did not possess inherently chiral geometries. Their observed differential optical response under an applied magnetic field was instead associated with the magneto-optical modification of electronic states, resulting in field-dependent interactions with circularly polarized light. Thus, this phenomenon is fundamentally different from optical activity originating from intrinsically twisted nanoparticles or hierarchically assembled chiral architectures. Nevertheless, coupling magnetic and optical components provides a promising route toward externally tunable polarization-dependent responses and may enable the development of magneto-optical nanomaterials for spin-dependent photonics, sensing, and catalytic applications [136]. Future studies should clearly distinguish such field-induced non-reciprocal optical phenomena from structural chirality while investigating possible coupling between magnetic, plasmonic, and genuinely chiral degrees of freedom.

### 4.8. Chirality Induced by Galvanic Displacement Reactions

Galvanic replacement reaction (GRR) is a versatile strategy for fabricating multicomponent chiral plasmonic nanomaterials by exploiting differences in the electrochemical potentials of two metals. During the reaction, a sacrificial metallic template is partially replaced by a second metal, producing alloyed or porous nanostructures while preserving the original morphology. This approach enables precise control over composition, architecture, and chiroptical properties [137]. Qin and co-workers demonstrated site-selective GRR by converting Ag nanocubes into concave Au–Ag alloy nanoframes through controlled HAuCl_4_ addition [138]. In another study, Huang and co-workers combined glancing angle deposition (GLAD) with GRR to transfer chirality from sacrificial Ag nanoparticles to porous Au–Ag alloy nanostructures [139]. The resulting nanoparticles exhibited enhanced and red-shifted circular dichroism, confirming efficient retention of chiral characteristics after alloy formation. The same strategy was extended to other alloy systems, including Ag–Pt, Ag–Pd, and Cu–Ag, and further adapted to fabricate ternary chiral nanostructures using Au as an intermediate scaffold [140,141]. Overall, GRR provides a robust route for producing porous multicomponent chiral alloys with tunable composition and optical activity. The combination of structural chirality, large surface area, and compositional versatility makes these nanomaterials attractive for applications in plasmonics, catalysis, sensing, and enantioselective reactions.

**Scheme 1:**
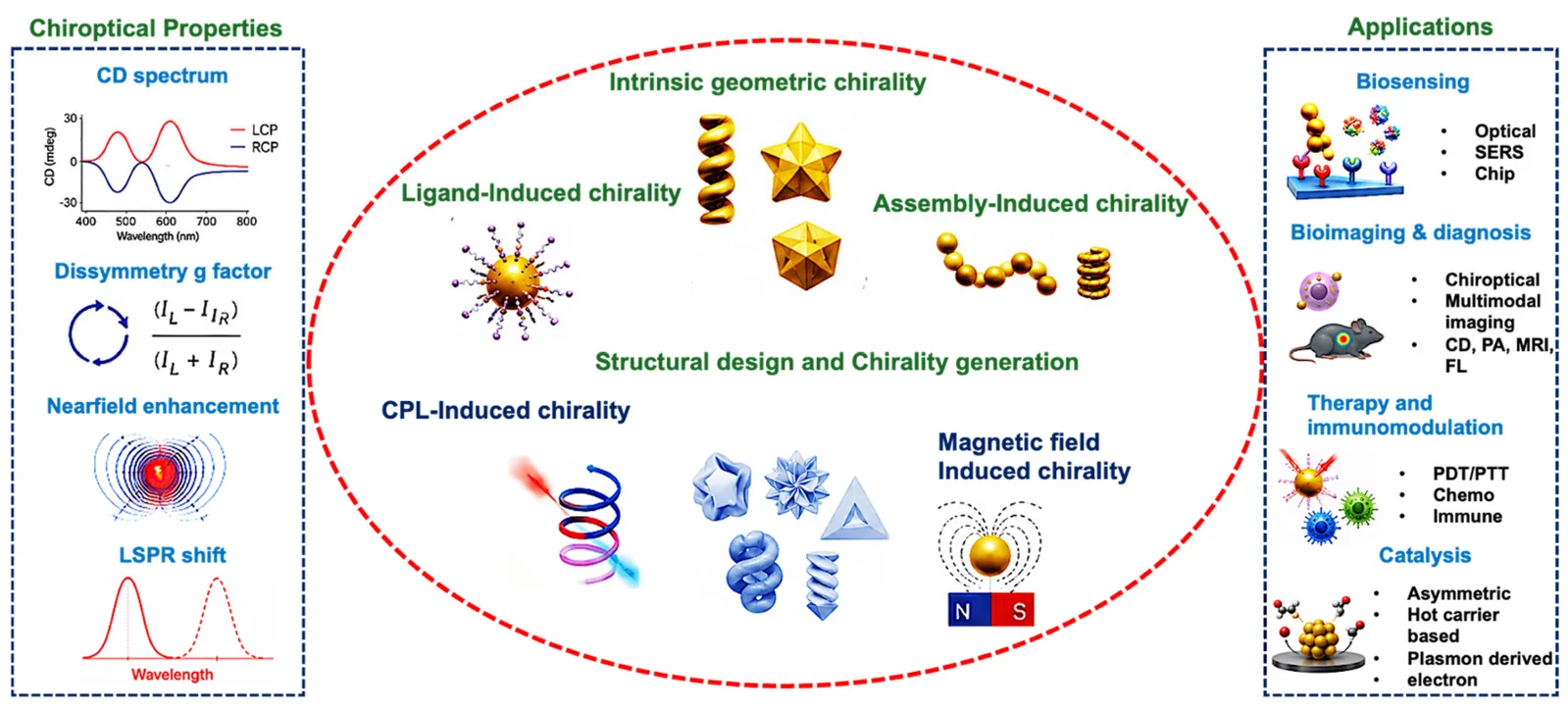
Integrated design–structure–property–application framework of chiral plasmonic nanomaterials. Plasmonic chirality can originate from ligand-induced surface interactions, intrinsic geometric asymmetry, hierarchical organization of plasmonic building blocks, or externally induced mechanisms. Control over nanoparticle morphology, asymmetric facets, interparticle spacing and orientation, hierarchical assembly, and composition regulates plasmonic coupling and interactions with circularly polarized light, thereby determining circular dichroism (CD), optical dissymmetry (g-factor), LSPR modulation, and near-field enhancement. These tunable chiroptical properties enable diverse functional applications in biosensing and enantiomeric recognition, bioimaging and therapy, immunomodulation, neurological applications, asymmetric catalysis, and related plasmonic technologies.

Overall, the functional performance of chiral plasmonic nanomaterials is governed by the close interplay between chirality origin, structural design, and the resulting chiroptical properties. Ligand-induced surface asymmetry, intrinsic geometric chirality, hierarchical organization, and externally regulated chirality generate distinct plasmonic responses that can be further tuned through nanoparticle morphology, interparticle spacing, orientation, and electromagnetic coupling. These structural features ultimately determine key optical characteristics, including circular dichroism, optical dissymmetry, LSPR behavior, and near-field enhancement. Scheme 1 summarizes these interconnected design–structure–property relationships and illustrates how they translate into functional applications in biosensing, biomedicine, catalysis, and related plasmonic technologies.

Despite the substantial progress in chiral plasmonic nanomaterial synthesis, each fabrication strategy offers distinct advantages and limitations in terms of structural control, reproducibility, scalability, and practical applicability. Ligand-induced approaches are among the simplest and most versatile strategies, as chiral molecules can directly transfer molecular asymmetry to plasmonic surfaces or selectively regulate crystal growth. These methods are generally compatible with solution-phase synthesis and provide considerable flexibility in surface chemistry; however, the resulting chirality can be highly dependent on ligand identity, surface coverage, binding stability, and reaction conditions, which may compromise long-term stability and reproducibility. Seed-mediated growth, in contrast, provides greater control over particle size, crystallographic facets, and three-dimensional morphology by separating nucleation from subsequent asymmetric growth. This approach has enabled highly chiral helicoidal and twisted nanostructures with strong chiroptical responses, but its performance is particularly sensitive to seed uniformity, ligand concentration, precursor addition, and growth kinetics, making batch-to-batch reproducibility and large-scale synthesis challenging. Template-assisted strategies offer the highest level of spatial and hierarchical control by organizing plasmonic building blocks using DNA, peptides, polymers, micelles, or inorganic chiral scaffolds. Such approaches enable precise regulation of interparticle spacing, orientation, and collective plasmonic coupling, but often require multistep fabrication, relatively expensive or complex templates, and carefully controlled assembly conditions, limiting scalability and practical implementation. Thus, no single synthetic strategy is universally optimal: ligand-induced methods provide simplicity and chemical versatility, seed-mediated growth offers strong control over intrinsic nanoparticle morphology, whereas template-assisted approaches provide superior control over higher-order organization. Selection of an appropriate fabrication route therefore requires balancing structural precision, chiroptical performance, reproducibility, scalability, and intended application, as summarized in Table 2.

## 5. Applications of Chiral Nanoparticles

### 5.1. Chiral Sensing Application

Chiral sensing is one of the most important applications of chiral nanoparticles. In recent years, a wide range of analytical techniques, including optical spectroscopy, CD, SERS, and reflectance spectroscopy, have been employed for the enantioselective detection of diverse chiral analytes using these nanostructures [142].

#### 5.1.1. Biosensing Using Optical Spectroscopy Studies

Chiral plasmonic nanoparticles, particularly those composed of gold (Au) and silver (Ag), have emerged as powerful platforms for enantioselective sensing because of their strong localized surface plasmon resonance (LSPR), high optical extinction, and exceptional chiroptical responses. These properties make them attractive for constructing highly sensitive sensing systems based on circular dichroism (CD). For instance, chiral plasmonic nanostructures have been employed as CD-active probes for the selective quantification of trace levels of Hg^2+^ ions in aqueous media [143]. Beyond single nanoparticles, coupled plasmonic architectures such as Au–Ag and Au–Au dimers exhibit distinct dual CD features corresponding to the plasmon resonances of both metals (approximately 399 nm for Ag and 526 nm for Au) [107]. Because these optical signatures are highly dependent on nanoscale geometry and interparticle coupling, they provide an effective strategy for detecting biomolecules, including nucleic acids and proteins.

**Figure 9:**
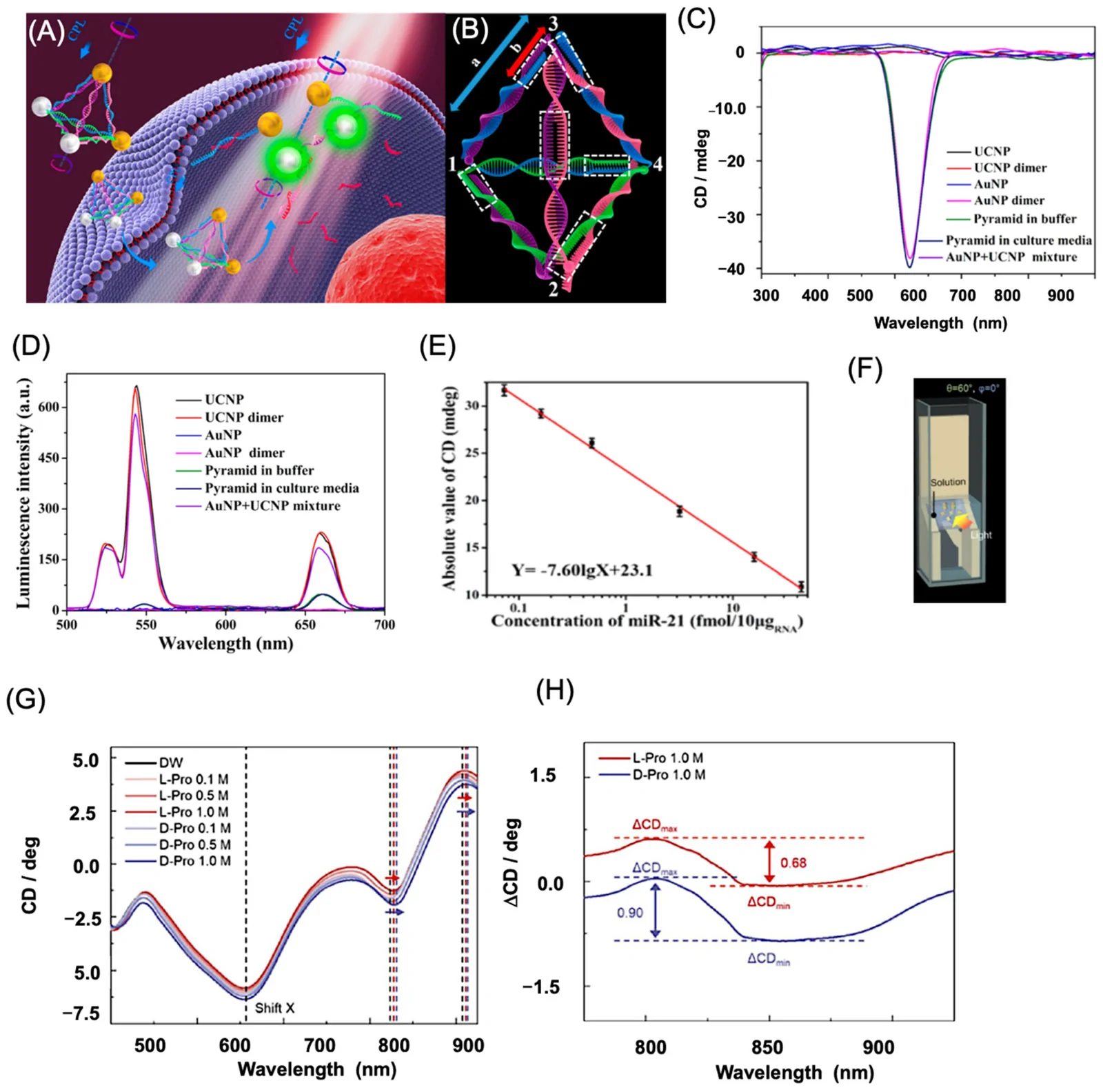
Chiral plasmonic nanostructures for enantioselective sensing and intracellular miRNA detection. (**A**) Schematic of an Au–UCNP pyramidal nanoassembly for intracellular miRNA sensing. (**B**) DNA framework directing target miRNA-mediated pyramidal assembly. (**C**) CD and (**D**) upconversion luminescence spectra of individual components and assembled nanostructures under 980 nm NIR irradiation. (**E**) Calibration of CD intensity at 521 nm against intracellular miR-21 concentration. (**F**) Experimental setup for enantiomer sensing using a 2D helicoid crystal. (**G**) CD spectra in water and L- or D-proline solutions, showing enantiomer-dependent modulation of collective CD bands. (**H**) Differential CD response (ΔCD) to L- and D-proline, demonstrating stronger modulation by D-proline. Panel (**A**–**E**) reproduced from [144]; Copyright 2015, American Chemical Society. Panel (**F**–**H**) reproduced from [124]; Copyright 2022, Nature publishing group.

A notable advancement was reported by Xu and co-workers, who introduced the first chiroplasmonic nanoassembly capable of monitoring intracellular microRNA (miRNA) in real time [144]. Their sensing platform relied on programmable self-assembly between gold nanoparticles and upconversion nanoparticles (UCNPs) directed by complementary DNA strands (Figure 9A,B) [144]. Hybridization of the target miRNA initiated the formation of well-defined pyramidal chiral nanostructures, bringing the Au and UCNP building blocks into proximity. This structural rearrangement simultaneously generated an intensified CD response and enhanced upconversion luminescence, allowing dual-mode detection of intracellular miRNA with excellent sensitivity and selectivity (Figure 9C,D). The DNA-templated pyramids exhibited a pronounced plasmonic CD signal centered near 521 nm together with strong luminescence in the 500–600 nm spectral region.

Importantly, both optical outputs increased linearly with miRNA concentration, enabling reliable quantitative analysis in living cells (Figure 9E). Among the two detection modes, the CD signal showed superior sensitivity, which was attributed to enhanced light–matter interactions within the chiral plasmonic framework and plasmon-assisted amplification of the intrinsic chirality of the DNA scaffold. Kwang-Tae Nam and co-workers reported a collective circular dichroism (CD)-based chiral sensing platform capable of highly sensitive enantioselective detection (Figure 9F) [124]. By exploiting pronounced variations in the collective CD response, the system enabled direct colorimetric visualization and quantitative analysis of molecular chirality. Beyond enantiomer discrimination, the platform was demonstrated for monitoring biologically relevant systems, including miR-21 and soluble N-ethylmaleimide-sensitive factor attachment protein receptor (SNARE) complexes. It also successfully tracked conformational changes associated with the interactions of neutravidin (NTV), vesicle proteins (Ves), and soluble VAMP2 (sVAMP2), highlighting its versatility for biosensing applications. For enantiomer recognition, two-dimensional helical crystal arrays were exposed to solutions of L- and D-proline using a colorimetric cell with an optical path length of 8.9 mm. The collective CD spectra exhibited distinct wavelength shifts and intensity variations depending on the molecular handedness, with D-proline producing a more pronounced red shift and stronger CD response than L-proline (Figure 9G,H). Furthermore, the sensor enabled simultaneous determination of both the L:D enantiomeric ratio and analyte concentration through experimentally established calibration relationships, allowing accurate quantification of molecular optical activity. Owing to its sensitivity toward structural changes, this collective CD sensing strategy holds considerable promise for investigating membrane protein folding and conformational dynamics, with future potential for probing structural changes in individual membrane proteins in situ.

Engineering the nanoparticle architecture further enhances chiroptical performance. Zhao and co-workers demonstrated that depositing additional Au or Ag shells onto plasmonic heterodimers significantly strengthened their optical activity while allowing precise tuning of the chiroptical response across the visible spectrum (400–600 nm) [145]. When combined with the exponential amplification provided by polymerase chain reaction (PCR), these core–shell chiral nanostructures enabled ultrasensitive DNA analysis with detection limits reaching the zeptomolar regime, offering considerable potential for early diagnosis of cancer, infectious diseases, and genetic mutations. The sensing platform achieved a linear analytical range from 160 zM to 1.6 pM, with a detection limit of approximately 11 pg mL^−1^. The remarkable sensitivity resulted from more efficient DNA hybridization in the nanoparticle assemblies together with enhanced plasmon–photon coupling. In addition to CD-based approaches, aggregation-induced colorimetric assays utilizing Au or Ag nanoparticles provide a simple and rapid alternative for real-time molecular detection. In these systems, analyte-induced nanoparticle aggregation causes distinct color changes that can be observed without sophisticated instrumentation [146]. As an example, Erhan Zor and colleagues developed a gold nanoparticle-based lab-in-a-syringe (LIS) platform for determining L-alanine levels in human serum [147]. The assay exhibited a detection limit of 0.77 mM, demonstrating the practicality of plasmonic colorimetric sensors for point-of-care and clinical diagnostic applications.

Lu et al. developed a simple and cost-effective polarization-directed growth strategy to fabricate highly ordered plasmonic chiral Au metamaterials with tunable handedness [148]. The self-aligned near-field guided anisotropic growth, producing nanostructures with strong circular differential scattering responses. By adjusting the alignment angle, the chiroptical response was optimized, enabling sensitive discrimination of molecular chirality. The platform detected L-cysteine at concentrations as low as 0.01 mg mL^−1^, while achiral molecules produced negligible dissymmetric signals, demonstrating excellent selectivity and reusability for chiral sensing. Beyond sensing, chiral Au nanomaterials have also been explored for enantioselective separation. Liu et al. incorporated L-cysteine-modified Au nanoparticles into graphene oxide membranes, where the nanoparticles acted as stable spacers to prevent sheet collapse while L-cysteine regulated the interlayer spacing [149]. The resulting membrane exhibited efficient size-selective transport and high enantioselectivity toward penicillamine racemates, achieving a chiral separation factor of 1.83 with excellent permeability.

#### 5.1.2. SERS-Based Enantioselective Sensors

**Figure 10:**
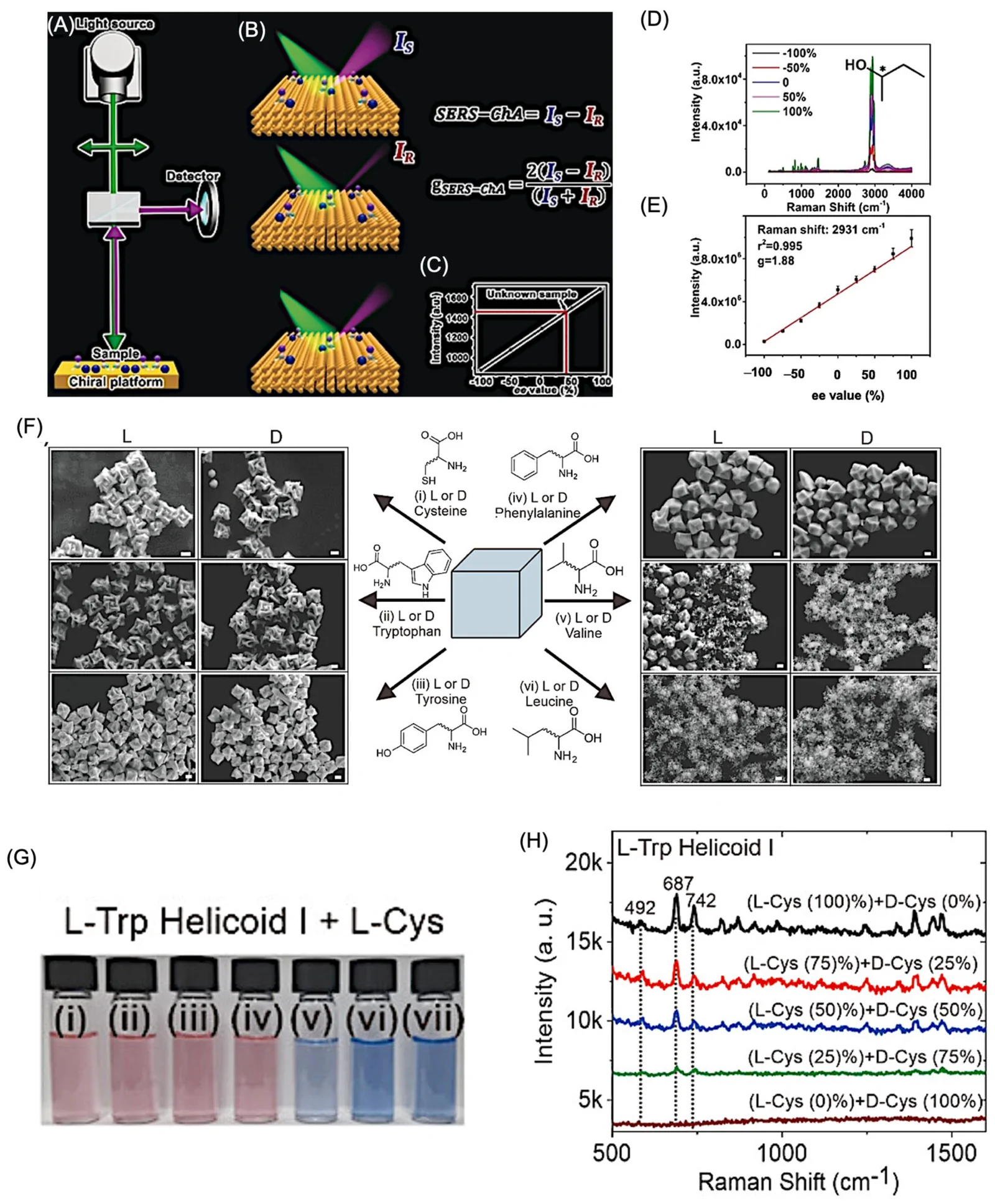
Chiral plasmonic SERS platforms for enantiomer discrimination and chiral sensing. (**A**) Schematic of a conventional Raman system integrated with a chiral plasmonic substrate. (**B**) SERS-based chiral anisotropy (SERS-ChA) measurement and determination of the dissymmetry (g) factor for enantiomeric analytes. (**C**) Calibration strategy for identifying absolute configuration and enantiomeric excess (ee) of unknown samples. (**D**,**E**) Representative Raman spectra and correlation between SERS peak intensity and ee values (−100% to +100%) for 2-butanol. * indicate the chiral center in 2-butanol. (**F**) FE-SEM images of chiral Au nanostructures synthesized in the presence of six different L- or D-amino acids (scale bar: 50 nm). (**G**) Colorimetric response of L-Trp Helicoid I after exposure to increasing concentrations of L-cysteine (0–10 µM, (i)–(vii) respectively). (**H**) Raman spectra of L-Trp Helicoid I in the presence of pure L-Cys, pure D-Cys, and L/D-Cys mixtures with different enantiomeric compositions. Panel (**A**–**E**) reproduced with permission from [128]; Copyright 2020, Wiley Publishing group. Panel (**F**–**H**) reproduced with permission from [64]; Copyright 2023, Wiley Publishing group.

Beyond monitoring bulk biomolecular interactions, future developments in chiral sensing may enable the direct observation of conformational transitions and folding dynamics of individual membrane proteins. In addition to CD, surface-enhanced Raman scattering (SERS) has emerged as another powerful analytical approach because it combines the molecular fingerprinting capability of Raman spectroscopy with the signal amplification provided by plasmonic nanostructures [150,151]. Exploiting this concept, Che and co-workers developed a surface-enhanced Raman scattering–chiral anisotropy (SERS-ChA) platform using chiral nanoparticle assemblies as plasmonic substrates (Figure 10A) [128]. Unlike conventional chiroptical techniques that rely on circularly polarized light, this method operates with linearly polarized illumination while still providing reliable discrimination between molecular enantiomers through differences in SERS intensity (Figure 10B). Calibration curves generated from the SERS-ChA response enabled quantitative determination of both enantiomer concentration and enantiomeric excess (ee) (Figure 10C). A notable feature of this sensing strategy is its consistently high anisotropy factor (g), with values typically ranging from 1.34 to 1.99 for a broad spectrum of chiral compounds. Evaluation of one hundred commercially available enantiomeric pairs demonstrated that the platform accurately differentiated molecules spanning diverse molecular weights, functional groups, and polarities (Figure 10D). Except for compounds possessing equivalent numbers of stereogenic centers, nearly all analytes could be quantitatively distinguished using the SERS-ChA response. Furthermore, the sensing performance remained stable under harsh experimental environments, including high ionic strength as well as strongly acidic and alkaline conditions. The platform also retained excellent selectivity in complex samples containing mixtures of chiral and achiral species, highlighting its potential for practical chemical and biological analyses.

Kumar et al. studied the influence of amino acid identity on the evolution of plasmonic chirality, which had also been systematically investigated using gold nanocube seeds as templates. Different amino acids directed the formation of distinct chiral morphologies, with tryptophan and cysteine producing the Helicoid I (432) architecture, whereas tyrosine favored the formation of Helicoid IV structures. In contrast, phenylalanine, valine, and leucine were unable to induce well-defined chiral nanostructures, highlighting the critical role of amino acid chemistry in directing chirality evolution (Figure 10E) [64]. The resulting tryptophan-mediated Helicoid I nanoparticles exhibited strong chiroptical activity and excellent enantioselective sensing toward L- and D-cysteine through both colorimetric and surface-enhanced Raman scattering (SERS) analyses (Figure 10F–H). Furthermore, the precisely engineered nanogaps (~24 nm) enabled highly sensitive discrimination of small chiral molecules, including epichlorohydrin, limonene, and 2-butanol, under non-polarized light illumination. These findings demonstrate that amino acid-directed growth provides an effective strategy for tailoring plasmonic chirality while expanding the application of helicoid nanostructures in chiral sensing and spectroscopic analysis. Wu et al. introduced a surface topographical engineering strategy to precisely regulate the surface morphology of intrinsically chiral gold nanocrystals by tuning the growth conditions with L- or D-cystine [152]. By controlling the degree of surface wrinkling, they generated nanocrystals with highly ordered, disordered, and less ordered chiral topographies, establishing a direct relationship between surface architecture and chiroptical performance. Notably, the ordered wrinkled surfaces created intense asymmetric electromagnetic “chiral hot spots,” which significantly enhanced plasmon-assisted enantioselective recognition. The engineered nanocrystals enabled highly sensitive discrimination of L- and D-penicillamine through plasmon-enhanced chiral sensing, demonstrating that nanoscale surface roughness is a critical structural parameter for amplifying localized chiral electromagnetic fields. This work highlights surface topographical engineering as an effective approach for designing next generation plasmonic nanomaterials with improved chiral sensing capabilities.

Beyond small molecules, chiral plasmonic nanostructures have shown considerable promise for biomedical sensing. Wang et al. engineered Pt@Au triangular nanorings using L/D-glutathione-directed growth to generate plasmonic nanostructures with strong chiroptical activity [152]. Owing to chirality-dependent plasmonic coupling, these nanorings selectively interacted with different aggregation states of amyloid-β (Aβ), enabling ultrasensitive discrimination of Aβ monomers and fibrils at femtomolar concentrations. Their ability to quantify Aβ directly in cerebrospinal fluid highlights the potential of chiral SERS platforms for the early diagnosis of Alzheimer’s disease and other protein aggregation-related neurodegenerative disorders. Alternative substrate designs have further expanded the scope of chiral SERS sensing. For example, asymmetric nanoporous gold bowls (NPGBs) integrated with electrochemical SERS generated intrinsic chiral surface sites that promoted stereoselective adsorption of analytes [153]. The application of an external electrochemical potential strengthened analyte–surface interactions, producing distinct Raman fingerprints for amino acid enantiomers such as tryptophan. Combined with density functional theory (DFT) calculations and chemometric analysis, this platform enabled accurate quantification of enantiomeric compositions while maintaining excellent reproducibility and label-free operation. More recently, Niu et al. developed chiral gold nanocrystals with precisely engineered chiral electromagnetic fields and embedded internal Raman standards to improve both signal stability and enantioselective sensitivity [154]. Theoretical analysis demonstrated that linearly polarized light could generate localized chiral near fields around the nanocrystals, producing significantly different Raman enhancement for opposite enantiomers. This design achieved stable discrimination of L- and D-phenylalanine and provides a robust framework for next-generation plasmonic SERS sensors with improved reliability and broader applicability in chiral molecular analysis. Overall, these studies demonstrate that engineering the morphology, surface chirality, and plasmonic near-fields of gold nanostructures substantially enhances SERS-based enantiomer recognition, offering highly sensitive and label-free platforms for applications ranging from biomarker detection to pharmaceutical and biomedical analysis. Although remarkable progress has been achieved, the sensing capabilities of chiral plasmonic nanomaterials continue to evolve. Future research should focus on designing nanostructures with stronger chiroptical amplification, higher signal-to-noise ratios, and improved selectivity in complex biological environments. Addressing these challenges will accelerate the development of highly sensitive and reliable chiral sensing platforms for applications ranging from biomedical diagnostics and pharmaceutical analysis to environmental monitoring.

#### 5.1.3. Chiral Nanomaterials as Electrochemical Sensors

Electrochemical sensing has emerged as a complementary strategy for chiral discrimination owing to its high sensitivity, rapid response, low cost, and simple instrumentation. Incorporating chiral nanomaterials into electrochemical platforms enhances enantioselective recognition by providing stereospecific adsorption sites, increasing the electroactive surface area, and facilitating efficient electron transfer. Chiral plasmonic nanostructures, metal nanoparticles, and hybrid nanocomposites have been successfully integrated with voltammetric and amperometric techniques for the selective detection of amino acids, pharmaceuticals, and other biologically relevant chiral molecules [155]. In particular, the combination of chiral surface engineering with electrochemical signal amplification has significantly improved detection sensitivity, enabling quantitative enantiomer analysis in complex samples. These advances highlight the growing potential of chiral nanomaterial-based electrochemical sensors for biomedical diagnostics, pharmaceutical quality control, environmental monitoring, and food safety.

Early demonstrations of chiral gold nanoparticle-based electrochemical sensors showed that penicillamine-functionalized Au nanoparticles could selectively recognize the enantiomers of 3,4-dihydroxyphenylalanine (DOPA) through chirality-dependent electron-transfer behavior, establishing the feasibility of electrochemical enantioselective sensing using intrinsically chiral plasmonic nanomaterials [156]. More recently, systematic investigations revealed that the enantioselective electrochemical performance of chiral Au nanoparticles is governed by key structural descriptors, including the chiroptical g-factor, surface geometry, and nanoparticle anisotropy. Establishing these structure–property relationships provides important design principles for optimizing chiral electrochemical sensors with enhanced selectivity and sensitivity [157]. Expanding this concept further, hierarchical step-rich Pt@Au nanostructures possessing abundant chiral surface motifs and intensified plasmonic near-fields were developed for plasmon-enhanced electrochemical recognition of penicillamine enantiomers and accurate determination of enantiomeric purity [158]. The same research group has demonstrated an enantioselective sensing platform for L- and D-penicillamine based on differential pulse voltammetry combined with chiral gold nanocrystals exhibiting controlled degrees of surface wrinkling [152]. By engineering highly disordered and plasmonically active surface features, the nanocrystals generated abundant chiral electromagnetic hot spots that significantly enhanced the discrimination between the two enantiomers. This study demonstrated that rational control over nanoscale surface morphology can markedly improve the sensitivity and selectivity of plasmon-assisted electrochemical chiral sensing.

Beyond conventional ligand-functionalized chiral electrodes, intrinsically chiral metallic nanostructures have recently emerged as a promising platform for enantioselective electrochemical biosensing. Unlike surface-modified systems, intrinsic chirality is embedded within the crystal architecture, providing enhanced structural stability, long-term chiroptical activity, and improved electrochemical performance. In this regard, Xu and co-workers developed intrinsically chiral Pd@AuPd alloy nanoparticles with a helical architecture for electrochemical biosensing [155]. The Pd-enriched surface significantly enhanced electrocatalytic activity while preserving the intrinsic chirality of the alloy, enabling highly sensitive detection of ascorbic acid and the simultaneous determination of dopamine and uric acid in complex biological samples. Compared with conventional chiral ligand-based systems, this strategy minimizes ligand desorption while improving both stability and sensing performance, highlighting the potential of intrinsically chiral metallic nanoarchitectures for next-generation electrochemical biosensors and multiplex bioanalytical applications. These studies collectively demonstrate that precise engineering of chiral surface architecture and electromagnetic near-fields plays a decisive role in improving electrochemical enantioselective recognition and provides a versatile strategy for next-generation chiral biosensing platforms.

Despite the remarkable sensitivity and enantioselectivity demonstrated by chiral plasmonic sensing platforms, several challenges remain before their broader analytical application. Chiroptical responses are highly sensitive to nanoparticle morphology, interparticle spacing, surface chemistry, and aggregation state, making batch-to-batch reproducibility and quantitative signal calibration particularly important. Complex biological samples may further introduce nonspecific interactions and matrix effects that alter plasmonic coupling and compromise selectivity. Future efforts should therefore focus on reproducible large-scale fabrication, standardized quantitative readouts, improved surface antifouling strategies, and integration with portable detection platforms. Combining chiroptical sensing with complementary modalities such as SERS, fluorescence, or microfluidic analysis may further improve sensitivity, molecular specificity, and practical applicability. Recent developments in optical chiral analysis have further expanded beyond conventional CD-based detection toward advanced nanophotonic platforms that exploit structured light, enhanced near-field interactions, metasurfaces, and optical forces for highly sensitive chiral discrimination and sorting. Emerging integration with artificial intelligence and machine-learning-assisted spectral analysis may further improve the identification and high-throughput analysis of complex chiral systems [142].

### 5.2. Chiral Nanoparticles in Photocatalysis

The convergence of plasmonic chirality with photocatalytic functionality has emerged as a promising direction in nanomaterials research [21]. The photocatalytic activity of plasmonic nanostructures originates from several interconnected light–matter interaction pathways rather than from LSPR enhancement alone. Upon resonant excitation, plasmonic nanoparticles can enhance photocatalysis through near-field electromagnetic enhancement, enhanced light scattering, hot-carrier generation and transfer, and plasmon-mediated energy transfer [51]. Strong localized electromagnetic fields increase optical absorption and electron–hole pair generation in neighboring semiconductor or molecular photocatalysts, whereas plasmonic scattering can increase the effective optical path length and improve light harvesting. Non-radiative plasmon decay can additionally generate energetic (“hot”) electrons and holes, which may cross a metal–semiconductor interface when their energies and interfacial band alignment are favorable, thereby participating directly in surface redox reactions or promoting charge separation [120,159]. However, carrier–carrier and carrier–phonon scattering rapidly redistribute plasmon-derived energy, and competition among carrier extraction, relaxation, and recombination strongly determines the overall photocatalytic efficiency. In hybrid plasmonic–semiconductor systems, spectral and spatial overlap between plasmonic modes and semiconductor excitonic transitions can additionally facilitate plasmon–exciton coupling and resonance energy transfer, modifying excitation generation and charge-separation pathways [160]. For chiral plasmonic photocatalysts, these mechanisms acquire an additional polarization-dependent dimension because opposite circular polarizations can interact differently with the chiral plasmonic modes, potentially producing asymmetric local fields, carrier populations, or substrate interactions. A quantitative understanding of these competing processes is therefore required to distinguish genuine plasmon-mediated stereoselective photocatalysis from thermal or conventional surface-catalytic effects.

**Figure 11:**
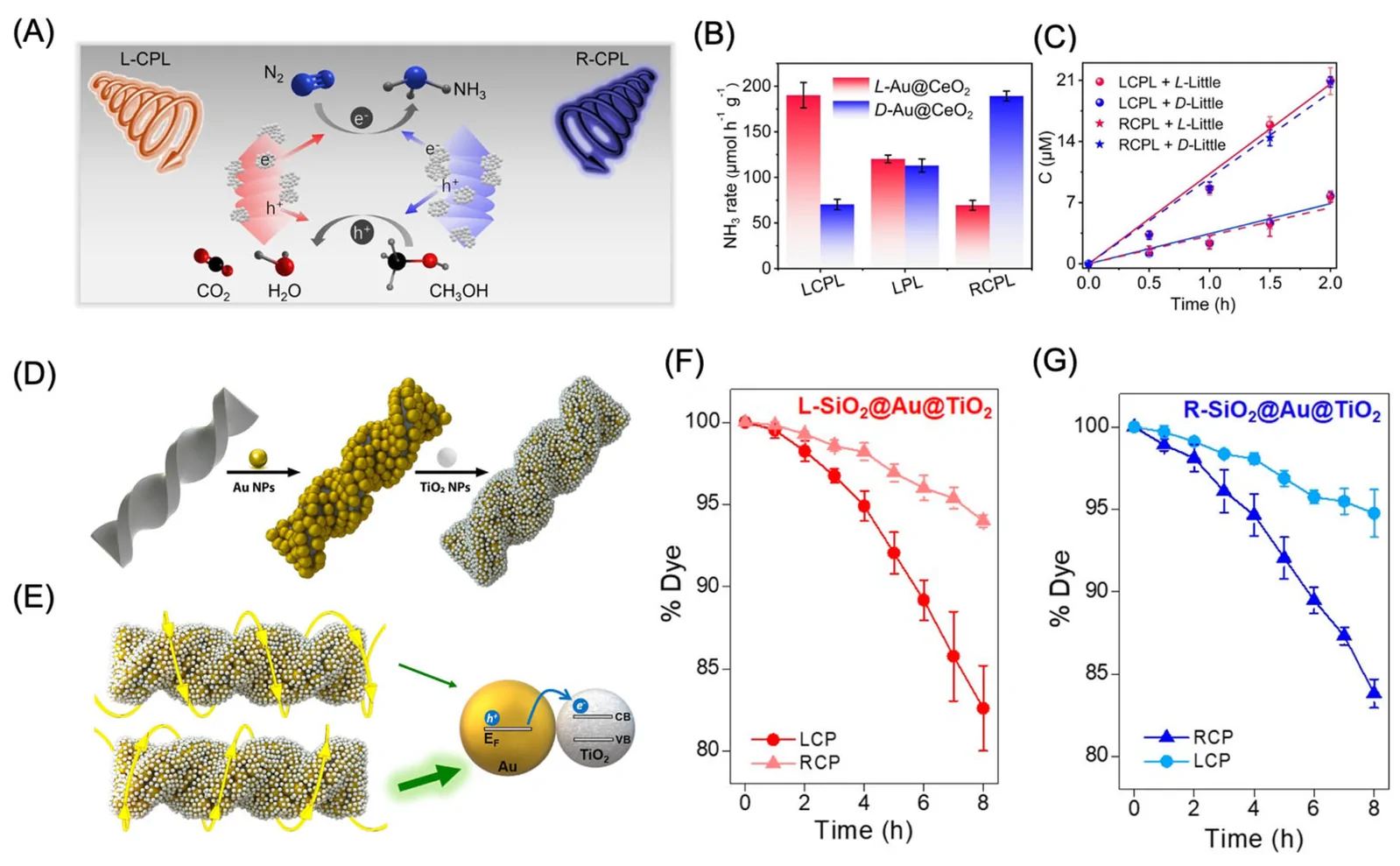
(**A**) CPL-driven nitrogen photofixation using L/D-Au@CeO_2_ helical nanorods (HNRs). (**B**) N_2_ fixation rates under LPL, LCPL, and RCPL irradiation. (**C**) Chirality-dependent NH_3_ generation by L- and D-Au@CeO_2_ HNRs under LCPL and RCPL (mean ± SD, *n* = 3). (**D**) Fabrication of chiral SiO_2_@Au@TiO_2_ photocatalysts using helical SiO_2_ nanoribbon templates. (**E**) Polarization-selective plasmonic excitation and hot-electron transfer from Au to TiO_2_. (**F**,**G**) CPL-dependent photocatalytic degradation of rhodamine B (RhB) by L- and R-SiO_2_@Au@TiO_2_ hybrid nanoribbons. Panel (**A**–**C**) Reproduced with permission from [161]; Copyright 2025, Wiley Publishing group. Panel (**D**–**G**) reproduced with permission from [120]; Copyright 2022, American Chemical Society.

Xu and colleagues developed helical Au@CeO_2_ nanorods through a facile wet-chemical synthesis, in which CeO_2_ was selectively deposited onto chiral gold nanorods, creating a spatially separated plasmonic–semiconductor interface (Figure 11A) [161]. This architecture significantly enhanced photocatalytic nitrogen fixation, achieving an activity approximately 50-fold higher than that of achiral Au nanorods (Figure 11B). Moreover, the photocatalytic response was strongly dependent on the handedness of both the nanostructure and the incident CPL. L-handed Au@CeO_2_ nanorods exhibited superior performance under left-handed CPL, whereas D-handed counterparts showed maximum activity under right-handed CPL, with nearly a threefold enhancement compared with irradiation using the opposite polarization (Figure 11C). The enhanced performance was attributed to chirality-dependent generation and separation of plasmon-induced hot charge carriers, highlighting the potential of chiral plasmonic heterostructures for polarization-controlled photocatalysis. In another study, Correa-Duarte and co-workers fabricated chiral plasmonic photocatalysts by directing the self-assembly of Au nanoparticles onto inorganic chiral TiO_2_ templates (Figure 11D,E) [120]. The resulting hybrid materials displayed pronounced polarization-sensitive photocatalytic behavior. Maximum catalytic activity was observed when the helicity of the incident CPL matched the intrinsic chirality of the plasmonic assembly (Figure 11F,G). This selective response originates from vortex plasmon modes that promote asymmetric excitation of hot electrons and holes, thereby enhancing charge-carrier generation and utilization. These findings demonstrate that coupling plasmonic chirality with semiconductor photocatalysts provides an effective route for manipulating photocatalytic reactions using circularly polarized light and opens new opportunities for enantioselective photochemistry and advanced chiral photocatalytic applications.

Surface engineering has enabled chiral gold nanomaterials to replicate the stereoselective catalytic behavior of natural enzymes, providing artificial nanozymes with high enantioselectivity. In one example, a chiral nanozyme consisting of cysteine-functionalized Au nanoparticles supported on expanded mesoporous silica (D-/L-Cys@AuNPs-EMSN) was developed, where AuNPs served as the catalytic sites, chiral cysteine imparted molecular recognition, and the mesoporous silica framework provided structural support [162]. The catalytic activity was evaluated using the enantiomeric substrate 3,4-dihydroxyphenylalanine (DOPA), revealing pronounced chirality-dependent substrate preference. Specifically, D-Cys@AuNPs-EMSN preferentially catalyzed the oxidation of L-DOPA, whereas L-Cys@AuNPs-EMSN exhibited enhanced activity toward D-DOPA. Kinetic analysis, activation energy measurements, and molecular dynamics simulations collectively indicated that selective hydrogen-bonding interactions between surface-bound cysteine and DOPA govern the observed enantioselectivity. These findings demonstrate the potential of chiral Au-based nanozymes as enzyme mimetics for asymmetric catalysis, with promising applications in the synthesis of chiral pharmaceuticals, agrochemicals, and other enantiomerically pure compounds. In a related study, Xu and co-workers designed chiral photocatalytic superparticles by first synthesizing quinidine-stabilized ZnS nanoparticles and subsequently directing their self-assembly into chiral architectures [163]. These assemblies were further integrated with glutathione-functionalized Au nanoparticles to produce hierarchical ZnS–Au hybrid superstructures. The resulting nanostructures displayed pronounced circular dichroism across the 200–550 nm spectral range, confirming their strong chiroptical characteristics. Their photocatalytic performance was evaluated using the enantiomers of tyrosine (Tyr), where L-ZnS–Au superparticles exhibited a clear preference for the photooxidation of L-Tyr over its D-counterpart. This enantioselective behavior highlights the influence of chiral plasmonic–semiconductor hybrid architectures on asymmetric photocatalysis and suggests that selective adsorption and spatial organization of substrate enantiomers at the catalyst surface play a key role in governing stereospecific reaction pathways. In addition to chiral plasmonic photocatalysts, chirally engineered metallic nanocrystals have also demonstrated significant potential in asymmetric heterogeneous photocatalysis. For example, chiral Pd nanocrystals synthesized using cinchonidine or S-proline as both shape-directing and chiral-inducing agents exhibited enhanced catalytic activity and enantioselectivity by eliminating conventional surface-capping ligands that often block active sites [97]. The in situ generated chiral Pd nanodendrites and nanocubes showed excellent stereoselective performance in asymmetric hydrogenation reactions, highlighting the importance of integrating intrinsic surface chirality with accessible catalytic interfaces. This strategy provides a versatile route for designing highly efficient chiral nanocatalysts with improved activity, selectivity, and stability, expanding the scope of chiral nanomaterials for asymmetric catalytic transformations.

For catalytic applications, chiral plasmonic nanomaterials provide an attractive interface between heterogeneous catalysis, enantioselective surface chemistry, and light-driven reactions. However, achieving high and reproducible enantioselectivity remains challenging because catalytic performance depends strongly on surface structure, ligand organization, active-site accessibility, and nanoparticle morphology. Structural reconstruction, ligand desorption, or aggregation under reaction conditions may further alter both catalytic activity and stereoselectivity. Future studies should establish clearer structure–activity–enantioselectivity relationships and evaluate catalyst stability, recyclability, substrate scope, and performance under practically relevant conditions. The integration of plasmonic excitation with asymmetric catalysis also offers opportunities to exploit hot carriers and localized electromagnetic fields for light-regulated and potentially more energy-efficient enantioselective transformations.

### 5.3. Biomedical Applications of Chiral Nanoparticles

#### 5.3.1. Tumor Bioimaging Applications

The integration of chirality into nanomaterial design has created new opportunities for improving the precision and sensitivity of tumor imaging [164]. Unlike conventional imaging probes, chiral nanomaterials exploit stereospecific interactions with biological systems to achieve selective tumor recognition while simultaneously enhancing the intrinsic imaging performance of the nanoplatform. The overall strategy relies on two complementary features: enantiomer-dependent interactions with the chiral biological microenvironment that promote preferential tumor accumulation, and chirality-induced modulation of the physicochemical properties of nanomaterials to amplify imaging signals [11]. Together, these characteristics enable higher imaging contrast, improved target specificity, and superior signal-to-background ratios for cancer diagnosis [23].

#### 5.3.2. Principles of Chiral Nanomaterial-Based Tumor Imaging

The effectiveness of chiral nanomaterials as imaging probes originates from their ability to recognize and respond to the stereochemical nature of biological systems [165]. Since biomolecules such as proteins, enzymes, membrane lipids, carbohydrates, and receptors possess intrinsic chirality, they interact differently with nanomaterials of opposite handedness. These stereoselective interactions influence cellular uptake, biodistribution, receptor affinity, and tumor retention, ultimately determining imaging performance.

One of the principal mechanisms underlying chiral tumor imaging is enantiomer-selective targeting. Through molecular “chiral matching,” nanomaterials with a specific handedness preferentially bind to tumor-associated receptors, resulting in enhanced accumulation within malignant tissues. For example, D-configured Fe_3_O_4_ superparticles exhibited stronger binding toward the CD47 receptor than their L-enantiomers, leading to improved tumor localization [166]. Likewise, R-configured Ag_2_Se/Yb/Er nanoparticles displayed dramatically higher affinity for the CD44 receptor than the corresponding S-enantiomers, demonstrating that subtle differences in nanoparticle chirality can substantially alter receptor recognition and in vivo targeting efficiency [167]. Besides improving tumor localization, chirality also enhances imaging performance by modifying the optical, electronic, and magnetic properties of nanomaterials. Rational control of nanoparticle chirality can regulate charge transfer, electronic energy levels, fluorescence emission, and magnetic relaxation behavior, resulting in stronger imaging signals and higher contrast. Consequently, chiral engineering provides a versatile platform that combines molecular recognition with signal amplification for highly sensitive tumor imaging.

#### 5.3.3. Magnetic Resonance Imaging Based on Chiral Nanomaterials

**Figure 12:**
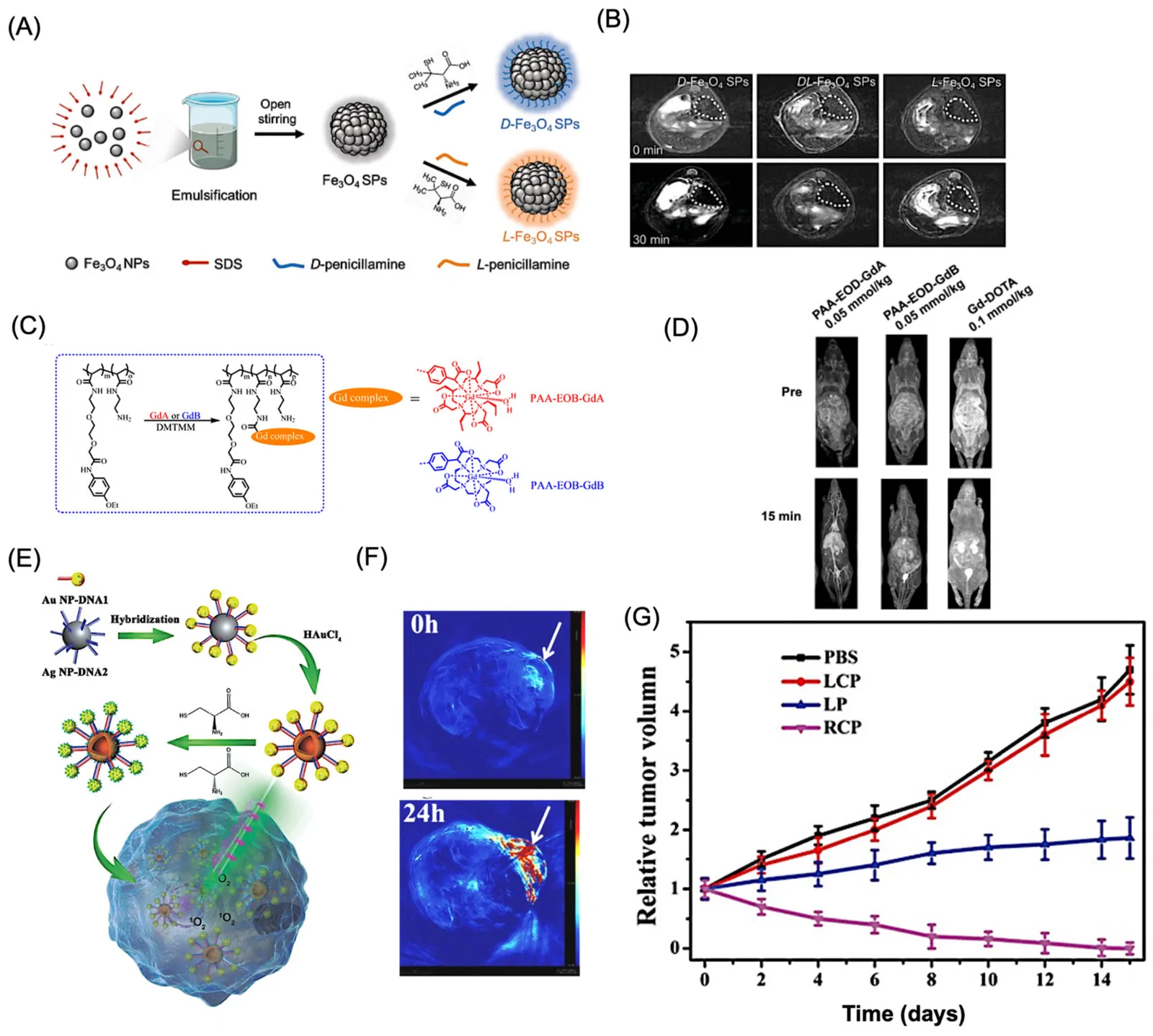
Representative examples of chiral nanomaterials for MRI and PAI-guided biomedical imaging. (**A**) Preparation of chiral Fe_3_O_4_ superparticles (SPs). (**B**) In vivo T_2_-weighted MRI and tumor-targeting evaluation of D-, DL-, and L-Fe_3_O_4_ SPs. (**C**) Synthesis and characterization of chiral gadolinium-based contrast agents PAA-EOB-GdA and PAA-EOB-GdB. (**D**) Magnetic resonance angiography following administration of PAA-EOB-GdA, PAA-EOB-GdB, or Gd-DOTA. (**E**) Self-assembled shell–satellite chiral nanoassembly for photodynamic therapy. (**F**) In vivo PA imaging of HeLa tumors following SS15-D-Cys administration. White arrowheads indicate the tumor region. (**G**) Relative tumor growth under LCP, LP, and RCP irradiation, demonstrating polarization-dependent therapeutic efficacy. Panel (**A**,**B**) reproduced with permission from [166]; Copyright 2023, Wiley publishers. Panel (**C**,**D**) reproduced with permission from [168], Copyright 2025, American Chemical Society. Panel (**E**–**G**) reproduced with permission from [170]; Copyright 2017, Wiley Publishers.

Magnetic resonance imaging (MRI) remains one of the most powerful diagnostic techniques owing to its excellent soft-tissue contrast, deep tissue penetration, and absence of ionizing radiation. Recent advances have demonstrated that introducing chirality into MRI contrast agents not only improves magnetic relaxation properties but also enhances tumor-targeting efficiency through stereoselective biological interactions. A representative example is the development of chiral Fe_3_O_4_ superparticles by Xu and co-workers [166]. Water-dispersible D- and L-Fe_3_O_4_ superparticles with an average diameter of approximately 80 nm exhibited distinct biological behaviors despite their identical chemical compositions (Figure 12A). Molecular docking studies revealed that D-enantiomers possessed approximately 2.3-fold higher affinity for the CD47 receptor than the corresponding L-form, resulting in significantly greater tumor accumulation in orthotopic breast cancer models. The hierarchical superparticle architecture further produced an exceptionally high transverse relaxivity (r_2_ = 498 mM^−1^ s^−1^), enabling markedly enhanced T_2_-weighted MRI contrast at the tumor site (Figure 12B). Chiral molecular engineering has also been successfully extended to gadolinium-based contrast agents. Dai and co-workers synthesized a polymeric MRI probe by incorporating ultra-stable chiral Gd-DOTA complexes into a poly(acrylic acid) (PAA) backbone decorated with ethoxybenzyl groups (EOB) [168]. This macromolecular platform exhibited a longitudinal relaxivity of 37.87 mM^−1^ s^−1^ at 0.5 T, representing an approximately twelve-fold improvement over clinically used Gd-DOTA. Binding to human serum albumin further increased the relaxivity to 43.23 mM^−1^ s^−1^. In rat VX2 liver tumor models, the chiral polymer generated prolonged vascular and tumor enhancement while requiring only one-eighth of the clinical dose of Gd-DOTA (Figure 12C,D). These findings demonstrate that chiral molecular engineering can substantially improve MRI sensitivity while simultaneously reducing the dosage of gadolinium-based contrast agents, thereby addressing limitations associated with conventional clinical MRI probes.

Li et al. reported chiral cobalt hydroxide nanoparticles that combine magnetic responsiveness with optical activity, enabling dual-mode ROS imaging through circular dichroism and magnetic resonance signals [169]. In the intracellular environment, ROS oxidized Co^2+^ to Co^3+^, producing a marked reduction in CD intensity together with an enhancement of the T1-weighted MRI signal. The D-aspartic acid-functionalized nanoparticles showed better analytical performance than the corresponding L-formulations, detecting intracellular ROS over a range of 0.673–612.971 pmol per 10^6^ cells. Their detection limits were 0.087 and 0.179 pmol per 10^6^ cells, corresponding to only 17% and 29% of those obtained with the L-enantiomeric counterparts. This stereoselective advantage was mainly associated with the greater cellular internalization of the D-configured nanoparticles. The same platform also supported quantitative evaluation of ROS in murine tumor tissues using combined MRI and fluorescence readouts, highlighting its potential for tumor microenvironment-responsive, multimodal molecular imaging [169].

#### 5.3.4. Photoacoustic Imaging Application of Chiral Nanoparticles

Photoacoustic imaging (PAI) has emerged as a powerful non-invasive and non-ionizing imaging modality that combines the high optical contrast of optical imaging with the deep penetration and spatial resolution of ultrasound, making it highly attractive for biomedical applications [171,172]. The rapid development of chiral nanomaterials has further expanded the capabilities of PAI by providing contrast agents with enhanced optical absorption, chirality-dependent interactions, and excellent tumor-targeting potential. Owing to their unique chiroptical and plasmonic properties, these nanomaterials have also been integrated with photothermal and photodynamic therapies to establish multifunctional theranostic platforms for imaging-guided cancer treatment. A notable example was reported by Xu and co-workers, who developed the first DNA-directed gold-core/silver-shell satellite-like chiral nanoassemblies for photoacoustic imaging and photodynamic therapy [170] (Figure 12E). The assemblies were functionalized with either L- or D-cysteine, producing strong plasmonic chiroptical responses in the visible region. Among the different formulations, the D-cysteine-modified nanoassemblies containing 15 nm Au cores and Ag shells exhibited the highest tumor-associated photoacoustic signal 24 h after intravenous administration (Figure 12F). Upon circularly polarized light (CPL) irradiation, these chiral nanostructures efficiently generated reactive oxygen species, resulting in significant tumor cell destruction through photodynamic effects (Figure 12G). This work demonstrated that DNA-programmed chiral plasmonic nanoassemblies can simultaneously provide enhanced photoacoustic contrast, real-time tumor visualization, and CPL-triggered therapeutic activity, highlighting their promise as multifunctional agents for PAI-guided cancer theranostics.

#### 5.3.5. NIR Fluorescence Imaging

**Figure 13:**
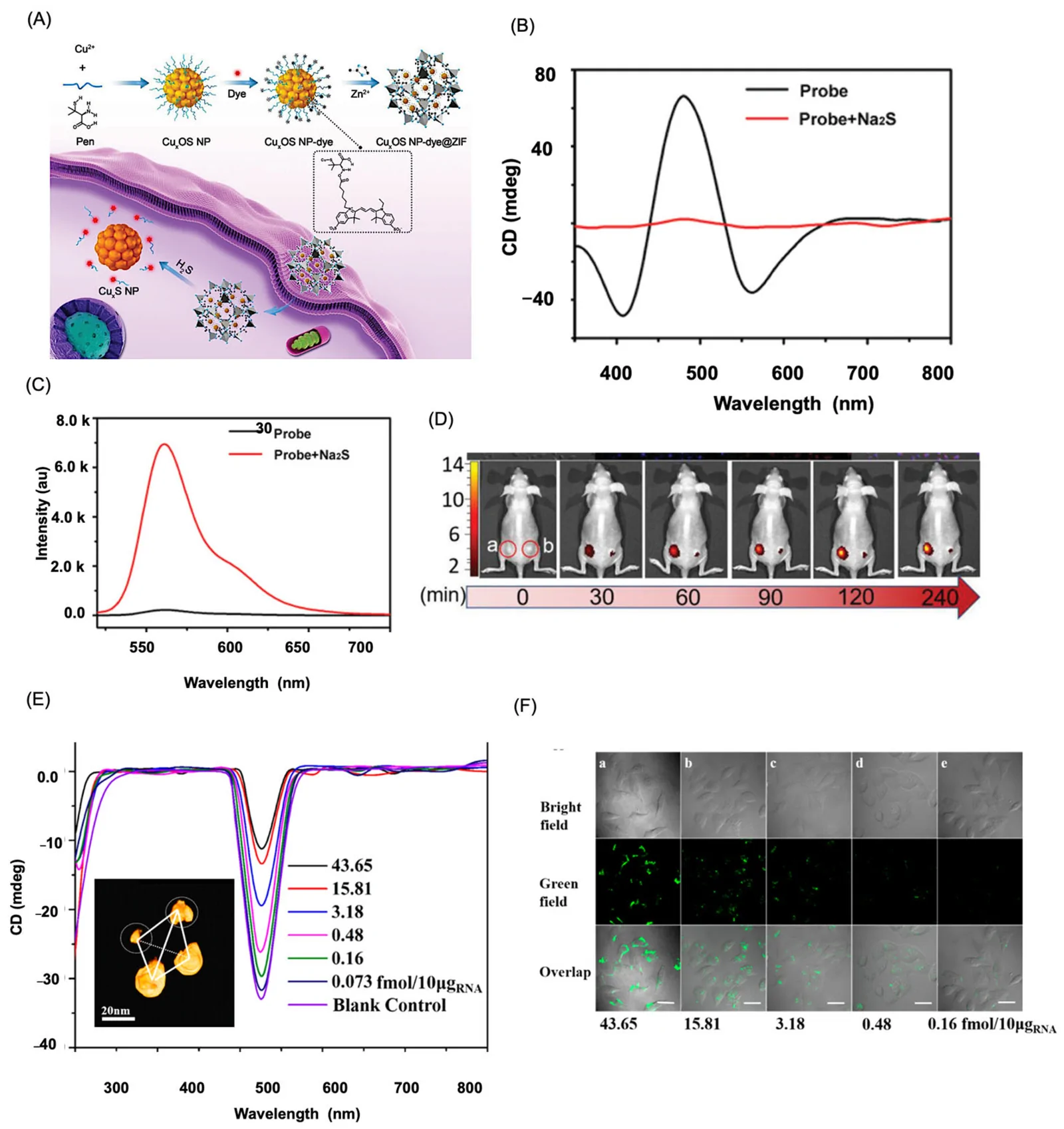
(**A**) CuxOS@ZIF-8 nanosensor for endogenous H_2_S detection. (**B**,**C**) CD and fluorescence responses of CuxOS-Cy3@ZIF-8 before and after Na_2_S treatment. (**D**) In vivo fluorescence imaging of endogenous H_2_S in tumor-bearing mice with (**a**) nanoprobe treatment and (**b**) PAG pretreatment. (**E**) CD spectra and (**F**) confocal fluorescence images of HeLa cells with different miR-21 levels: (**a**,**b**) miR-21-transfected, (**c**) non-transfected, and (**d**,**e**) antisense miR-21-transfected cells. Scale bar = 20 μm. Inset in (**E**): cryo-TEM tomographic reconstruction of the chiral pyramidal nanostructure. Panel (**A**–**D**) reproduced with permission from [174], Copyright 2020, Wiley Publishers. Panel (**E**,**F**) reproduced with permission from [144], Copyright 2016, American Chemical Society.

The integration of chirality with fluorescence imaging has led to the development of multifunctional nanoprobes capable of simultaneously providing optical visualization and chiroptical information [164]. By combining CD with fluorescence or upconversion luminescence (UCL), these dual-modal platforms offer enhanced sensitivity and improved analytical reliability compared with conventional fluorescence probes [173]. Such systems typically exploit stimulus-responsive changes in chiral assembly, allowing biological events to be monitored through concurrent variations in fluorescence intensity and chiroptical signals. One representative strategy employs metal–organic framework (MOF)-based chiral nanostructures for intracellular biomarker detection. For example, Cu_x_OS@ZIF-8 nanoparticles incorporating the fluorescent dye Cy3 were designed as dual-responsive probes for hydrogen sulfide (H_2_S) (Figure 13A) [174]. In the absence of H_2_S, the fluorescence of Cy3 remained quenched within the nanostructure. Upon exposure to H_2_S, decomposition of the probe released the dye, producing a concentration-dependent fluorescence recovery accompanied by a gradual decrease in the CD signal (Figure 13B,C). This complementary optical response enabled both quantitative determination of intracellular H_2_S and fluorescence imaging in living systems (Figure 13D).

Upconversion nanoparticles (UCNPs) have also emerged as attractive building blocks for chiral bioimaging because of their excellent photostability, deep tissue penetration, and low background autofluorescence [175]. DNA-programmed self-assembly has been utilized to fabricate chiral nanopyramids capable of real-time intracellular microRNA sensing. The intact nanostructure generated a pronounced CD response while suppressing UCL emission. Hybridization with the target microRNA triggered disassembly of the nanopyramid, leading to disappearance of the chiroptical signal together with restoration of UCL intensity [144]. This switchable dual-readout mechanism enabled sensitive intracellular microRNA detection by combining quantitative CD analysis with fluorescence imaging (Figure 13E,F). Similar design principles have subsequently been extended for monitoring reactive oxygen species (ROS) in cultured cells and tumor-bearing animal models. Another approach combines UCNPs with MOF-based hybrid architectures to construct ratiometric chiral imaging probes. Encapsulation of UCNP cores within NiS_x_-containing ZIF-8 shells produced nanocomposites exhibiting distinct CD activity together with wavelength-dependent modulation of upconversion emission [176]. Oxidative stimulation selectively altered the green emission while maintaining the red emission, allowing ROS concentrations to be quantified through fluorescence intensity ratios in parallel with CD signal changes. This dual-modal strategy provided highly sensitive imaging of intracellular hydrogen peroxide and enabled dynamic visualization of oxidative stress in vivo. Beyond molecular imaging, chiral fluorescence probes have also demonstrated considerable potential for pathogen detection. Chiral heterodimers assembled from antibody-functionalized gold nanoparticles and polymyxin-B-modified UCNPs generated amplified CD signals accompanied by quenched fluorescence [177]. Differences in the interaction of polymyxin B with antibiotic-sensitive and resistant Escherichia coli strains resulted in distinct optical responses, enabling discrimination between bacterial phenotypes and non-invasive visualization of infection in animal models.

Near-infrared II (NIR-II, 1000–1700 nm) fluorescence imaging has emerged as one of the most promising optical imaging modalities because of its superior tissue penetration, reduced light scattering, and minimal background autofluorescence compared with conventional visible and NIR-I imaging. The incorporation of chirality into NIR-II nanoprobes further enhances imaging performance by coupling stereoselective biological recognition with optimized optical emission. Consequently, chiral nanomaterials have attracted considerable attention as tumor-targeted imaging agents capable of improving both probe accumulation and imaging sensitivity. One of the earliest demonstrations of this concept was reported using chiral Ag_2_S quantum dots, where the influence of nanoparticle chirality on in vivo biodistribution was systematically investigated [178]. In an orthotopic renal tumor model, D-enantiomeric Ag_2_S quantum dots accumulated substantially more efficiently within tumors than their L-counterparts, reaching approximately 12.3% injected dose per gram of tissue after 24 h, which corresponded to nearly 2.1-fold greater tumor retention. These findings established that nanoparticle chirality plays a decisive role in regulating tumor homing and in vivo pharmacokinetics, providing direct evidence that stereochemical design can significantly improve the targeting efficiency of NIR-II imaging probes. Further improvements in imaging performance have been achieved by integrating chirality with rare-earth engineering. Xu and co-workers developed ultrasmall chiral Ag_2_Se nanoparticles co-doped with Nd^3+^, Yb^3+^, and Er^3+^ ions, producing a highly efficient NIR-II fluorescent probe. Rare-earth incorporation dramatically increased fluorescence brightness, yielding an emission intensity approximately 146-fold greater than that of undoped Ag_2_Se nanoparticles while shifting the emission maximum to 1550 nm, a wavelength well suited for deep-tissue imaging [167]. In addition to optical enhancement, nanoparticle chirality strongly influenced biological recognition. The R-enantiomer displayed exceptionally strong binding toward the CD44 receptor, exhibiting a dissociation constant of only 0.9 nM and nearly 927-fold higher affinity than the S-enantiomer. This stereoselective interaction translated into markedly improved tumor accumulation in vivo, enabling high-contrast NIR-II fluorescence imaging with a signal-to-noise ratio approaching 18 and the visualization of tumor margins with a spatial resolution of approximately 0.3 mm, thereby facilitating the detection of small metastatic lesions.

The application of chiral plasmonic materials for bioimaging applications is represented in Table 3. From a mechanistic perspective, chirality-dependent biological responses cannot be attributed solely to differences in nanoparticle internalization. Following exposure to biological fluids, chiral nanomaterials interact with proteins and other biomolecules to form a dynamic protein corona that defines their biological identity. Surface chirality may influence the composition, orientation, and conformation of adsorbed proteins through stereoselective interactions, thereby modifying receptor recognition, membrane interactions, and subsequent endocytic pathways. These nano–bio interactions can also influence immune recognition, since adsorption of opsonins may promote macrophage uptake and systemic clearance, whereas dysopsonin-rich coronas can reduce recognition by the mononuclear phagocyte system and potentially prolong blood circulation. Consequently, differences in protein-corona formation may contribute to chirality-dependent cellular uptake, immunogenicity, biodistribution, and circulation kinetics. Establishing systematic relationships between nanoparticle handedness, corona composition, cellular recognition, and in vivo pharmacokinetics therefore remains important for understanding and translating the biological effects of chiral plasmonic nanomaterials.

### 5.4. Chiral Nanoparticles for Therapy

The unique physicochemical and biological characteristics of chiral nanomaterials have established them as promising platforms for a wide range of therapeutic applications [164]. Beyond their distinctive chiroptical properties, these nanomaterials exhibit stereochemistry-dependent interactions with biological systems, enabling selective cellular uptake, differential protein adsorption, receptor-specific recognition, and immune modulation. Such enantioselective behavior significantly influences their biodistribution, pharmacological activity, and therapeutic efficacy, providing opportunities to improve treatment precision while minimizing off-target effects. Recent advances have demonstrated that the biological activity of chiral nanomaterials extend far beyond imaging and biosensing. Their ability to regulate cellular signaling pathways, oxidative stress, inflammatory responses, and immune activation has enabled the development of innovative therapeutic strategies for diverse diseases. Chirality-dependent nanoplatforms have shown considerable promise in enhancing cancer therapy through improved tumor targeting and therapeutic selectivity, while also offering new opportunities for the treatment of neurodegenerative disorders and age-associated diseases [25]. Increasing evidence further indicates that stereochemical engineering can modulate biological responses at the molecular level, providing mechanistic insights into how chirality governs interactions with proteins, membranes, and intracellular pathways.

#### 5.4.1. Chiral Nanomaterials for Phototherapy and Radiotherapy

Phototherapy has emerged as an important non-invasive strategy for cancer treatment, with nanomaterials playing a central role in improving therapeutic efficiency through enhanced light absorption and selective tumor accumulation. Among the available approaches, photothermal therapy (PTT) eradicates tumor cells by converting absorbed light into localized heat, whereas photodynamic therapy (PDT) relies on the light-triggered generation of reactive oxygen species (ROS) to induce oxidative damage and apoptosis [34]. The introduction of chirality into nanomaterials has further expanded the potential of these therapies by enabling polarization-dependent light–matter interactions that enhance phototherapeutic performance [190,191].

Unlike achiral nanostructures, chiral nanomaterials interact preferentially with circularly polarized light (CPL), resulting in more efficient optical energy harvesting than under linearly polarized light. This unique characteristic can significantly improve ROS production and phototherapeutic efficacy [164]. A representative example is provided by chiral supramolecular nanostructures constructed from cysteine-derived building blocks, which exhibited markedly enhanced singlet oxygen generation when irradiated with CPL of matching handedness [170]. Under these conditions, ROS production was approximately two-fold higher than that obtained under linearly polarized illumination. The enhanced photodynamic activity translated into pronounced chirality-dependent cytotoxicity, where D- and L-configured nanostructures achieved maximum therapeutic efficacy under right- and left-handed CPL, respectively. Importantly, efficient tumor cell destruction was achieved at relatively low photosensitizer concentrations, and complete tumor regression was observed in animal models following CPL-guided treatment.

**Figure 14:**
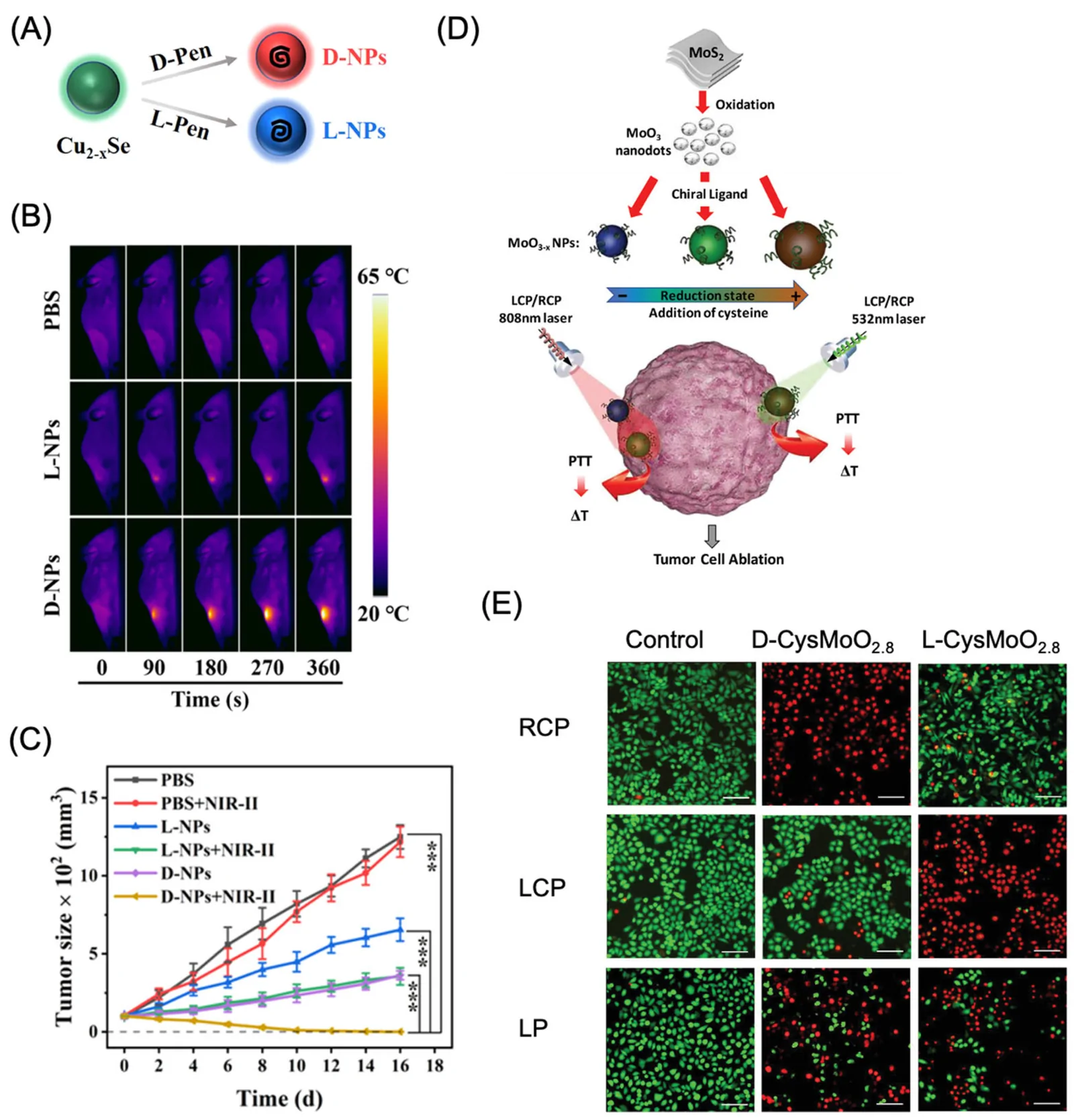
Chiral nanomaterials for photothermal cancer therapy. (**A**) Synthesis of D-/L-penicillamine-functionalized Cu_2−x_Se nanoparticles. (**B**) Representative tumor thermal images following different treatments. (**C**) Tumor growth curves showing treatment efficacy (*** *p* < 0.001). (**D**) Preparation of chiral Cys-MoO_3−x_ nanoparticles and their application in CPL-mediated photothermal therapy. (**E**) LIVE/DEAD images of HeLa cells treated with D- or L-Cys-MoO_2.8_ under RCP, LP, or LCP irradiation (808 nm, 1 W cm^−2^, 15 min). Scale bar = 100 μm. Panel (**A**–**C**) reproduced with permission from [192], Copyright 2021, American Chemical Society. Panel (**D**,**E**) reproduced with permission from [194]; Copyright 2020, Wiley Publishing group.

A representative example was reported by Liu and co-workers, who developed D- and L-penicillamine-modified Cu_2−x_Se nanoparticles for combined chemodynamic and photothermal therapy under second near-infrared (NIR-II) laser irradiation (Figure 14A) [192]. Compared with the L-enantiomer, the D-configured nanoparticles produced stronger therapeutic responses, primarily due to their higher photothermal conversion efficiency and enhanced ROS generation (Figure 14B,C). In addition, localized photothermal heating accelerated the release of therapeutic species, promoting hydroxyl radical production and resulting in more effective tumor cell destruction. Beyond direct tumor ablation, chiral nanomaterials have also been employed to facilitate postoperative tissue repair. Wang et al. developed a supramolecular hydrogel by incorporating polydopamine nanoparticles into an L-phenylalanine-derived gel matrix [193]. Under near-infrared irradiation, the nanoparticles generated sufficient heat to eliminate residual cancer cells, whereas the chiral hydrogel provided an extracellular matrix-like environment that supported fibroblast migration and tissue regeneration. This multifunctional system simultaneously suppressed tumor recurrence and accelerated wound healing following surgery. Li et al. introduced cysteine-functionalized MoO_3−x_ nanoparticles possessing intrinsic chiral architectures generated through metal-to-ligand charge transfer interactions (Figure 14D). These nanoparticles exhibited broad visible-light absorption and efficiently converted absorbed light into heat, leading to rapid temperature elevation during laser irradiation. Combined with their low cytotoxicity and efficient cellular internalization, the chiral MoO_3−x_ nanostructures represent promising and economical photothermal agents (Figure 14E) [194]. Despite these promising results, further investigations are required to evaluate the long-term stability, pharmacokinetics, and tumor accumulation efficiency of these supramolecular assemblies under physiologically relevant conditions before clinical translation can be considered.

Beyond light-mediated therapy, chirality has also been explored to improve the efficacy of radiotherapy [195,196]. Chiral gold nanoclusters have recently attracted attention as radiosensitizers capable of amplifying radiation-induced oxidative stress. In one study, D-configured Au nanoclusters demonstrated superior radiosensitizing activity compared with their L-enantiomeric counterparts [197]. The enhanced therapeutic response was attributed to improved intracellular distribution, which promoted ROS generation, DNA double-strand damage, cell-cycle arrest, and apoptotic cell death following X-ray irradiation. Although these ultrasmall nanoclusters produced encouraging antitumor outcomes, their extremely small dimensions (~2 nm) may result in rapid renal clearance and limited circulation time, potentially restricting tumor retention. Consequently, optimizing nanoparticle size, surface chemistry, administration protocols, and dosing strategies will be essential for maximizing their therapeutic benefits.

#### 5.4.2. Chiral Nanomaterials for Cancer Immunotherapy

Cancer immunotherapy has transformed modern oncology by stimulating the host immune system to recognize and eradicate malignant cells while establishing long-term immune memory [198]. However, its clinical efficacy is often compromised by poor antigen presentation, inadequate activation of immune effector cells, limited infiltration of cytotoxic lymphocytes, and the immunosuppressive tumor microenvironment. Chiral nanomaterials have recently emerged as a new class of immunomodulatory platforms because stereochemical engineering not only improves nanoparticle delivery but also regulates immune-cell recognition, activation, and cytokine signaling, thereby enhancing antitumor immunity [70].

**Figure 15:**
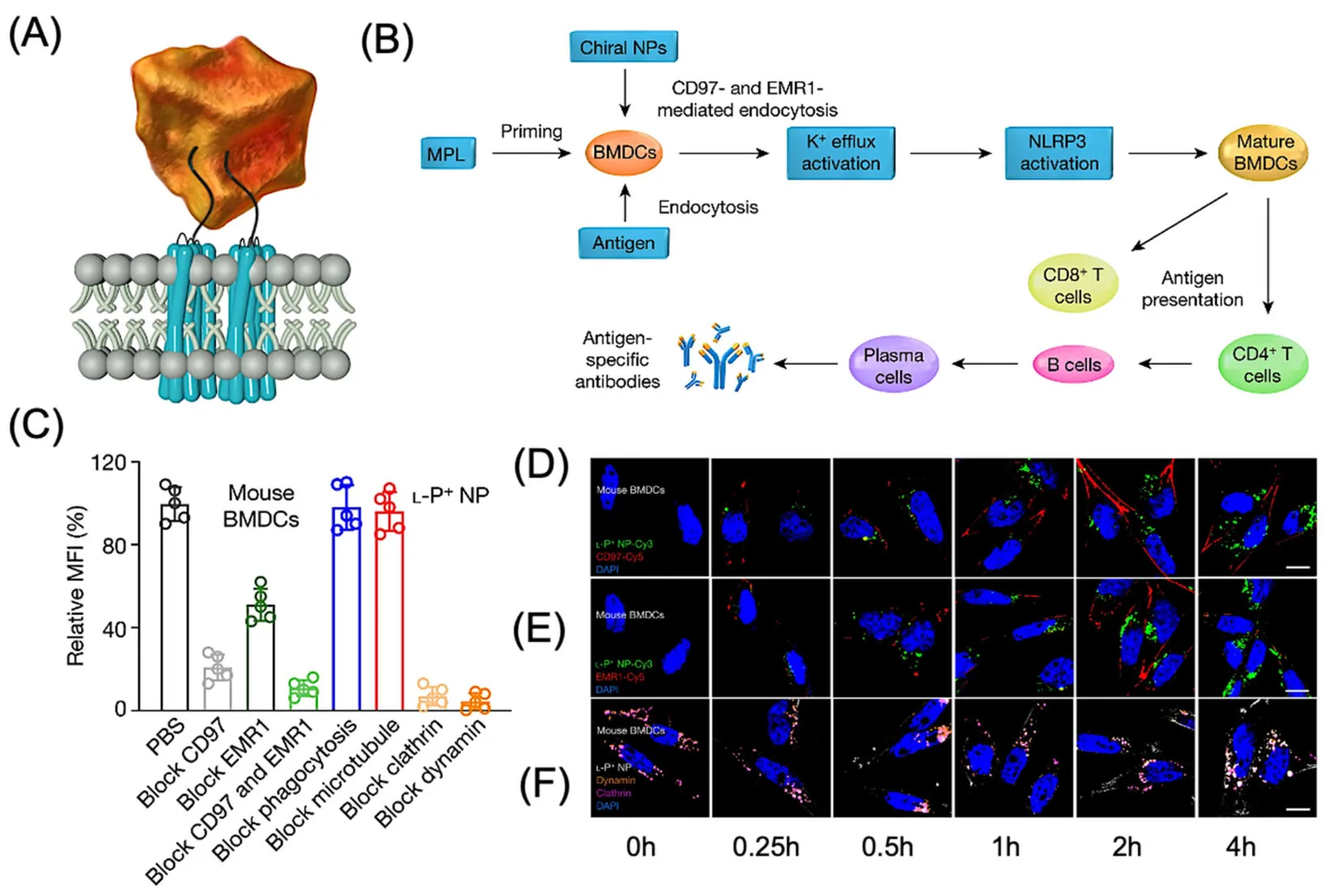
Chiral nanoparticles for immunotherapy applications. (**A**) Interaction of chiral nanoparticles with EGF-like domains (blue color) of adhesion G protein-coupled receptors (aGPCRs, grey color). (**B**) Proposed mechanism of chiral nanoparticle-mediated immune activation. (**C**) Relative MFI of BMDCs following receptor and endocytosis inhibition, indicating CD97/EMR1-mediated, clathrin- and dynamin-dependent internalization. (**D**,**E**) Time-dependent confocal images of BMDCs showing L-P^+^ nanoparticle colocalization with CD97 and EMR1 receptors (0–4 h); DAPI (blue), CD97/EMR1-Cy5 (red), and L-P^+^-Cy3 (green). (**F**) TPL images showing intracellular trafficking and colocalization with dynamin (orange) and clathrin (pink); L-P^+^ nanoparticles (white) and nuclei/DAPI (blue). Scale bars = 10 μm. Reproduced with permission from [130]. Copyright 2022, Nature Publishing group.

A pioneering study by Xu and co-workers demonstrated that chiral gold nanoadjuvants can simultaneously enhance innate and adaptive immune responses in a chirality-dependent manner (Figure 15A,B) [130]. These plasmonic nanostructures, synthesized under circularly polarized light, possessed exceptionally strong chiroptical activity, and their immunostimulatory efficacy increased with the optical anisotropy (*g*) factor. Mechanistic studies revealed that left-handed nanoadjuvants exhibited stronger interactions with G-protein-coupled receptors, including CD97 and EMR1, leading to enhanced cellular uptake, accelerated dendritic-cell maturation, and increased secretion of pro-inflammatory cytokines. Activation of the NLRP3 inflammasome further promoted antigen presentation and immune-cell activation. Consequently, left-handed nanoadjuvants generated substantially stronger antigen-specific antibody responses and, in tumor models, significantly enhanced infiltration of CD8^+^ cytotoxic T lymphocytes while activating natural killer (NK) cells through increased expression of NKG2D, granzyme B, and perforin, ultimately producing superior antitumor efficacy (Figure 15C,D).

Beyond regulating immune activation, chirality has also been exploited to improve nanoparticle pharmacokinetics and tumor delivery. D-configured MoS_2_/CoS_2_ nanozymes displayed prolonged circulation and enhanced tumor accumulation through stereoselective interactions with biological components [199]. Following tumor localization, these nanozymes efficiently generated reactive oxygen species, reprogramming immunosuppressive M2 tumor-associated macrophages into pro-inflammatory M1 macrophages and thereby amplifying local antitumor immune responses. Similarly, left-handed helical artificial antigen-presenting cells (aAPCs-L) minimized protein corona formation through stereochemical mismatch with endogenous serum proteins, substantially reducing phagocytic clearance, extending blood circulation beyond 24 h, and improving tumor accumulation, which ultimately resulted in complete melanoma regression.

Chiral engineering has also demonstrated considerable promise in therapeutic vaccination and oncolytic immunotherapy. Chen and co-workers reported that L-configured peptide hydrogels elicited significantly stronger antitumor immunity than the corresponding D-enantiomers, effectively suppressing immunosuppressive microenvironment formation and reducing both primary tumor growth and postoperative recurrence [200]. Likewise, Li and co-workers developed virus-inspired chiral oncolytic particles (VOPs), in which D-enantiomeric particles exhibited superior tumor-selective cytotoxicity and approximately five-fold greater oncolytic activity than the clinically used peptide LTX-315 [201]. Treatment with D-VOPs markedly increased intratumoral CD8^+^ cytotoxic T-cell infiltration and induced robust systemic antitumor immunity through chirality-dependent regulation of tumor recognition, cellular interactions, biodegradation resistance, and immune activation. Collectively, these studies demonstrate that chirality serves as a powerful molecular regulator of cancer immunotherapy by simultaneously enhancing nanoparticle delivery, controlling immune-cell signaling, remodeling the tumor microenvironment, and strengthening adaptive immune responses. These advances establish stereochemical engineering as a promising strategy for developing next-generation nanoadjuvants, therapeutic cancer vaccines, and immunotherapeutic nanomedicines with improved precision and therapeutic efficacy.

#### 5.4.3. Chiral Nanomaterials for Neurodegenerative Disease Therapy

Neurodegenerative disorders, including Alzheimer’s disease (AD) and Parkinson’s disease (PD), are characterized by progressive neuronal loss resulting from protein misfolding, oxidative stress, mitochondrial dysfunction, chronic neuroinflammation, and impaired neuronal regeneration. Despite considerable advances in pharmacological interventions, effective disease-modifying therapies remain elusive. Chiral nanomaterials have recently emerged as promising therapeutic platforms because their stereochemistry-dependent interactions with biological macromolecules enable selective recognition of pathological proteins, modulation of oxidative stress, and regulation of neural cell behavior.

One of the most extensively investigated applications of chiral nanomaterials is the inhibition of pathological protein aggregation. Chiral gold nanoparticles functionalized with glutathione demonstrated enantiomer-dependent binding toward amyloid-β (Aβ_42_), with D-enantiomeric nanoparticles exhibiting significantly stronger affinity than their L-counterparts [202]. The enhanced interaction effectively suppressed Aβ fibrillation, improved blood–brain barrier penetration, and produced superior cognitive recovery in Alzheimer’s disease models. Similar stereoselective effects have been reported for D-penicillamine-modified Fe_x_Cu_γ_Se nanoparticles, which not only inhibited Aβ aggregation but also promoted the disassembly of preformed amyloid fibrils under near-infrared irradiation through enhanced ROS generation. Likewise, chiral Cu_x_Co_γ_S superparticles effectively inhibited α-synuclein aggregation and facilitated fibril dissociation in Parkinson’s disease models, highlighting the broad applicability of chirality-guided protein recognition for treating protein-misfolding disorders [203].

Beyond regulating protein aggregation, chiral nanomaterials have also shown considerable promise in alleviating oxidative stress, a central pathological feature shared by most neurodegenerative diseases. Nanozymes possessing superoxide dismutase-, catalase-, glutathione peroxidase-, and peroxidase-like activities efficiently eliminated intracellular reactive oxygen species, thereby reducing neuronal oxidative damage and improving cognitive function in Parkinson’s disease models [204]. Morphology-dependent catalytic activity has also been demonstrated in Mn_3_O_4_ nanostructures, where nanorod-shaped particles exhibited superior ROS-scavenging capability compared with other morphologies [205]. In addition, antibody-functionalized chiral gold nanoparticles selectively eliminated senescent microglia through apoptosis, reducing α-synuclein accumulation and mitigating neuroinflammation [206]. These studies illustrate the potential of chiral nanozymes to simultaneously regulate oxidative stress, cellular senescence, and inflammatory signaling within the diseased brain.

Chiral nanomaterials have further emerged as powerful tools for neural regeneration by directing stem-cell fate through stereoselective mechanobiological signaling. Chiral plasmonic gold films modified with enantiomeric penicillamine exhibited opposite effects on neuronal cell adhesion and differentiation under circularly polarized light, demonstrating that surface chirality can regulate neuronal development [207]. More recently, DNA-programmed chiral nanoassemblies have been employed to precisely control neural stem-cell differentiation [208]. Under circularly polarized illumination, these nanostructures generated chiroplasmonic mechanical forces through interactions with cytoskeletal proteins, activating neuron-specific genetic programs and promoting efficient neuronal differentiation. Following transplantation into Alzheimer’s disease models, the differentiated neurons integrated into damaged brain tissue and significantly improved cognitive performance, suggesting that light-responsive chiral nanomaterials may provide an innovative strategy for neuronal regeneration.

Despite these encouraging advances, several translational challenges remain. Most reported chiral nanotherapeutics require direct intracranial administration because their relatively large dimensions limit efficient penetration across the blood–brain barrier. Future research should therefore prioritize the development of ultrasmall, biodegradable, and actively targeted chiral nanoplatforms capable of crossing the blood–brain barrier following systemic administration while maintaining high stereoselectivity and long-term biosafety. Addressing these challenges will be critical for translating chiral nanomedicine from proof-of-concept studies toward clinical treatment of neurodegenerative diseases.

#### 5.4.4. Chiral Nanomaterials for Age-Related Diseases

The progressive accumulation of senescent cells is recognized as a major contributor to aging and numerous age-associated disorders through persistent secretion of pro-inflammatory factors, mitochondrial dysfunction, and impaired tissue regeneration. Consequently, the selective elimination of senescent cells (senolysis) has emerged as a promising therapeutic strategy to delay age-related functional decline while preserving healthy tissues [209]. In this context, chiral nanomaterials offer unique advantages because their stereochemistry-dependent cellular interactions enable selective recognition, targeted intracellular delivery, and controlled therapeutic activation. One of the earliest demonstrations of chiral nanomaterial-mediated senolysis was reported using mitochondria-targeted core–shell spiky nanorods (CSNRs) functionalized with triphenylphosphonium and β_2_-microglobulin antibodies [210]. These nanoplatforms selectively accumulated within senescent cells, where near-infrared (NIR) irradiation induced mitochondrial membrane disruption and reactive oxygen species (ROS) generation, triggering apoptosis. In addition to direct phototherapeutic effects, activation of immune responses further promoted senescent cell clearance, resulting in improved hair regeneration and enhanced physical performance in doxorubicin-induced aging models. Despite these encouraging outcomes, the optimization of in vivo stability, targeting specificity, and long-term biosafety remain essential for future clinical translation. Chirality has also been exploited to improve the efficiency of senescent-cell targeting through stereoselective cellular uptake. Photo- and magnetically responsive chiral Cu_x_Co_γ_S nanoparticles demonstrated pronounced enantiomer-dependent internalization, with D-configured nanoparticles exhibiting approximately 2.5-fold greater uptake than their L-counterparts because of stronger interactions with cell membrane components [211]. Combined NIR irradiation and alternating magnetic field stimulation generated synergistic oxidative stress and cytoskeletal disruption, leading to activation of caspase-3-mediated apoptosis and efficient elimination of senescent cells in vivo. These findings highlight how stereochemical engineering can enhance both nanoparticle delivery and therapeutic efficacy through multimodal stimulation.

Beyond phototherapeutic approaches, programmable chiral nanostructures have also enabled stimulus-responsive senolytic drug delivery. DNA-bridged tetrahedral nanoplatforms consisting of upconversion nanoparticles surrounded by gold nanoparticles were engineered for the controlled release of granzyme B [212]. Following selective recognition of senescent cells via β_2_-microglobulin antibodies, NIR irradiation disrupted the DNA framework, releasing granzyme B to initiate apoptosis. Simultaneously, fluorescence signal conversion provided real-time monitoring of therapeutic activity, enabling image-guided senolytic treatment. Application of this platform effectively reduced senescent-cell burden and reversed aging-associated phenotypes in both accelerated-aging and doxorubicin-induced senescence models.

Collectively, these studies demonstrate that chirality provides a versatile strategy for engineering next generation senolytic nanomedicines by enhancing cellular selectivity, therapeutic precision, and multimodal treatment efficacy. Future efforts should focus on developing biodegradable chiral nanoplatforms with improved targeting specificity, long-term biosafety, and clinically applicable delivery routes to facilitate translation for the treatment of aging and age-associated disorders. The application of chiral plasmonic materials for age and neurodegenerative diseases is represented in Table 4.

Although chiral plasmonic nanomaterials have demonstrated considerable potential across bioimaging, therapeutic delivery, phototherapy, immunomodulation, and neurological applications, their biomedical translation remains at an early stage. Importantly, the challenges are application dependent. For imaging and image-guided applications, reproducible chiroptical responses, signal stability in complex biological environments, tissue penetration, and quantitative interpretation remain important considerations. Therapeutic applications additionally require precise control over dose, biodistribution, treatment selectivity, and long-term fate of the plasmonic components. In immunotherapeutic applications, a deeper understanding of how nanoparticle handedness influences protein corona formation, immune-cell recognition, inflammatory signaling, and adaptive immune responses is required. Similarly, applications involving neurodegenerative diseases face additional barriers associated with blood–brain barrier transport, neuronal targeting, neurotoxicity, and long-term retention.

More broadly, the biological effects of nanoscale chirality remain insufficiently understood, and systematic comparisons between opposite enantiomeric nanostructures are still required to establish reliable relationships between handedness and cellular uptake, biodistribution, therapeutic efficacy, and toxicity. Future studies should therefore integrate rigorous physicochemical and chiroptical characterization with pharmacokinetic, immunological, and long-term safety evaluation in clinically relevant models. The development of biodegradable or efficiently clearable chiral nanostructures, biomimetic surface engineering, disease-specific targeting, and multimodal image-guided therapeutic platforms may help bridge the gap between the unique properties of chiral plasmonic nanomaterials and their eventual clinical translation.

## 6. Conclusions and Future Perspectives

Chiral nanomaterials have emerged as one of the most rapidly advancing areas of nanoscience, bridging chemistry, materials science, catalysis, and biomedicine. This review has highlighted recent progress in the rational synthesis of chiral nanostructures through molecular induction, chiral templating, circularly polarized light (CPL), magnetic-field-assisted assembly, and self-assembly strategies, demonstrating how precise control over nanoscale chirality enables unique optical, catalytic, and biological functions. These advances have significantly expanded the application landscape of chiral nanomaterials, ranging from asymmetric catalysis and enantioselective sensing to bioimaging, disease diagnosis, cancer therapy, immunotherapy, and regenerative medicine.

A key advantage of chiral nanomaterials lies in their ability to exploit stereospecific interactions that are inaccessible to conventional achiral nanostructures. In catalytic systems, chirality provides new opportunities for asymmetric photocatalysis, enantioselective electrocatalysis, and biomimetic nanozymes with enhanced selectivity and catalytic efficiency. In the biomedical field, stereochemical engineering has enabled selective recognition of proteins, nucleic acids, and cell-surface receptors, resulting in improved biosensing, targeted imaging, precision drug delivery, immune modulation, and disease therapy. Increasing evidence indicates that nanoparticle chirality not only governs molecular recognition but also regulates intracellular signaling, protein adsorption, cellular uptake, immune activation, and therapeutic outcomes, establishing chirality as a powerful design principle for next-generation nanomedicine.

Despite these remarkable achievements, several challenges continue to limit the broader application and clinical translation of chiral nanomaterials. The scalable synthesis of nanostructures with well-defined chirality, high optical anisotropy, and excellent batch-to-batch reproducibility remains difficult. Furthermore, the fundamental mechanisms governing chirality-dependent interactions with complex biological systems are still incompletely understood. Current studies often focus on phenomenological observations, whereas detailed molecular-level investigations combining advanced spectroscopy, cryogenic electron microscopy, molecular dynamics simulations, artificial intelligence-assisted structural prediction, and multi-omics analyses are required to establish robust structure–activity relationships. Standardized evaluation protocols are also essential to enable meaningful comparisons between different chiral nanoplatforms.

From a biomedical perspective, future efforts should prioritize the development of biodegradable, biocompatible, and clinically translatable chiral nanomaterials with predictable pharmacokinetics, long-term biosafety, and efficient clearance. Improving blood circulation, active tissue targeting, blood–brain barrier penetration, and tumor-specific accumulation while minimizing off-target toxicity will be critical for successful clinical implementation. In parallel, integrating chirality with multifunctional nanoplatforms capable of combining imaging, biosensing, targeted drug delivery, immunotherapy, phototherapy, gene regulation, and catalytic therapy is expected to accelerate the emergence of personalized theranostic systems. The incorporation of responsive elements activated by circularly polarized light, magnetic fields, ultrasound, or endogenous biochemical stimuli may further enable precise spatiotemporal control over therapeutic activity.

Looking ahead, several exciting research directions are anticipated to shape the next generation of chiral nanotechnology. The development of intrinsically chiral inorganic nanocrystals, programmable supramolecular assemblies, chiral metal–organic frameworks, and hybrid organic–inorganic architectures will provide unprecedented opportunities for tailoring optical and catalytic properties. Expanding the role of chirality in photoelectrochemical catalysis, artificial photosynthesis, enzyme-inspired catalysis, quantum nanomaterials, and intelligent nanosystems represents another promising frontier. Moreover, combining machine learning with high-throughput materials discovery and automated synthesis could substantially accelerate the rational design of chiral nanostructures with optimized physicochemical and biological performance.

Future advances in chiral nanomaterials are expected to focus on multifunctional platforms that integrate molecular sensing, bioimaging, targeted drug delivery, and therapeutic functions within a single nanostructure. Stimuli-responsive chiral systems capable of adapting to disease-specific microenvironments (e.g., pH, ROS, or enzymatic activity) will further improve targeting precision and therapeutic efficacy. In addition, chirality-mediated immune modulation represents a promising strategy for enhancing immunotherapy. The integration of artificial intelligence, molecular simulations, and data-driven material design is also anticipated to accelerate the rational development and optimization of next-generation chiral nanomedicines, facilitating their translation toward precision diagnostics and personalized therapy.

Overall, chiral nanomaterials have evolved from a fundamental scientific curiosity into a versatile platform with broad implications for catalysis, sensing, imaging, and precision medicine. Continued interdisciplinary collaboration among chemists, materials scientists, engineers, computational researchers, and clinicians will be essential to address the remaining scientific and translational challenges. With continued advances in stereochemical engineering, mechanistic understanding, and scalable manufacturing, chiral nanomaterials are expected to play an increasingly important role in sustainable catalysis, intelligent diagnostics, and next-generation precision therapeutics.

## Figures and Tables

**Table 1:** Representative chiral plasmonic nanostructures, chiral ligands, optical activity, and representative applications.

Nanostructure	Chiral Induction Strategy	Typical *g*-Factor	Representative Application	Ref.
Au Octahedra	L/D-Cys, GSH	0.20	Polarization control, chiral optics	[63]
Au Nanocubes	L/D-Cys, Trp, Tyr	0.03, 0.018 and 0.011	SERS, plasmonics	[64]
Au Octahedra	peptide ligands (g-Glu-cys/Cys-Gly)	0.02	Chiral optics	[65]
Au Octahedra	ssDNA	0.04	Chiral optics	[66]
Au Decahedra	CYP/GSH	—	Chiral plasmonics	[67]
Au Nanorods	L/D-Cys, 4-ATP	0.03–0.1	CPL generation, optical modulation	[68,69]
Au Nanotriangles	CYP, Cys	0.08–0.44	Cancer immunotherapy	[70,71]
Au Nanodiscs	GSH	0.10	Chiral optics	[72]
Rhombicuboctahedra	CYP	0.01	Plasmonic chirality	[73]
Trisoctahedra	Cys	0.02	Enantiomer discrimination	[74]

Where Cys: Cysteine, GSH: Glutathione, Trp: Tryptophan, Gly: Glycine, CYP: Cysteine–Phenylalanine, ssDNA: Single-stranded DNA.

**Table 2:** Correlation between chirality origin, structural characteristics, synthetic strategies, and functional attributes of representative chiral plasmonic nanomaterials.

Chirality Origin	Structural Characteristics	Synthetic Strategy	Mechanism/Principle	Representative Systems	Key Advantages	Major Limitations	Representative Applications
Surface/atomic-level chirality	Asymmetric ligand–metal coordination; chiral surface environment; asymmetric surface electronic states	Chiral ligand functionalization/direct induction	Chiral molecules adsorb or coordinate to metal surfaces, transferring molecular chirality through ligand–metal electronic interactions	Au, Ag, Cu, Pd NPs and nanoclusters	Simple; versatile surface chemistry; readily tunable ligand composition	Ligand desorption; environmental sensitivity; chirality may depend strongly on surface coverage	Enantioselective recognition, sensing, asymmetric catalysis, biomedical applications
Intrinsic geometric chirality	Twisted/helicoidal morphology; asymmetric high-index facets; nanoscale gaps and protrusions	Seed-mediated asymmetric growth	Enantioselective adsorption of chiral ligands on specific crystallographic facets alters growth kinetics and generates asymmetric 3D morphologies	Au helicoids, Au nanorods, Au@Ag structures	Strong CD and high *g*-factors; excellent morphological control; tunable optical response	Sensitive to seed quality, ligand concentration, and reaction conditions; reproducibility and scale-up remain challenging	SERS, enantiomer discrimination, sensing, bioimaging, photothermal applications
Hierarchical/assembly induced chirality	Helical, crossed, pyramidal, or other asymmetric arrangements of plasmonic building blocks	DNA-programmed assembly	Sequence-specific hybridization positions nanoparticles with nanoscale precision, generating chiral plasmonic coupling	AuNP/AuNR assemblies, Au–Ag assemblies	Programmable geometry; precise interparticle spacing; strong collective CD	High cost; multistep preparation; sensitivity to ionic strength and temperature	Biosensing, chiral optics, bioimaging, photonic systems
Hierarchical/assembly induced chirality	Biomolecule-directed helical or supramolecular nanoparticle organization	Peptide/protein/biomolecule-guided assembly	Intrinsically chiral biomolecules direct nanoparticle nucleation, growth, or spatial organization	Au and Ag nanostructures	Biocompatible; mild aqueous synthesis; biomimetic	Lower structural reproducibility; environmentally sensitive	Biosensing, bioimaging, drug delivery, catalysis
Hierarchical/assembly induced chirality	Nanoparticles arranged along pre-existing helical or twisted scaffolds	Template-assisted synthesis/assembly	Soft or hard chiral templates guide nanoparticle nucleation, deposition, or organization	Au/Ag on silica helices, micelles, polymers, biotemplates	Good structural control; tunable architecture; enhanced plasmonic coupling	Multistep fabrication; template preparation/removal; scalability and cost	Chiral sensing, catalysis, optical materials, photonic applications
Collective/hierarchical chirality	Ordered 2D/3D chiral nanoparticle arrays and films	Nanofilm fabrication/self-assembly	Long-range organization and interparticle plasmonic coupling amplify the chiroptical response	Au, Ag, Cu chiral films	Large-area optical response; enhanced CD; suitable for device integration	Maintaining uniform handedness over large areas; fabrication complexity	Optical sensing, polarization control, photonic devices
Optical chirality	Light-directed asymmetric growth or morphology; switchable handedness	CPL-assisted synthesis	Circularly polarized light provides a chiral optical field that directs asymmetric nucleation, growth, or plasmon-mediated deposition	Au and hybrid plasmonic nanostructures	Direct optical control; tunable/switchable chirality; molecular inducer may not be required	Specialized optical setup; low throughput; limited scalability	Chiroptics, optical switching, sensing, information processing
Magnetic-field-induced magneto-optical response (non-geometric)	Magnetic–optical hybrid nanostructures; intrinsic geometric chirality is not required	Integration of magnetic domains with semiconductor or plasmonic nanostructures followed by external magnetic-field modulation	External magnetic field breaks time-reversal symmetry and modifies electronic states and polarization-dependent optical transitions, producing non-reciprocal magneto-optical/chiroptical responses rather than structural chirality	Fe_3_O_4_-containing hybrid nanostructure heteronanorods; magnetic–plasmonic hybrid nanostructures	External and potentially reversible control; integrates magnetic and optical functions	Requires magnetic components; relatively weak effects in many systems; limited material library	Magneto-optics, responsive materials, imaging, spin-dependent photonics
Transferred/intrinsic geometric chirality	Porous, hollow, concave, or multimetallic chiral architectures	Galvanic replacement reaction	Controlled replacement of a sacrificial metal preserves or transfers structural chirality while altering composition and morphology	Au–Ag, Ag–Pt, Ag–Pd, Cu–Ag structures	Tunable composition and LSPR; high surface area; multifunctionality	Difficult control of replacement kinetics, final composition, and structural chirality	Catalysis, sensing, imaging, phototherapy, enantioselective reactions

**Table 3:** List of chiral nanomaterials for bioimaging applications.

Biomedical Modality	Role of Chirality	Representative Chiral Nanomaterials	Representative Applications	Ref
MRI	Chirality enhances molecular recognition and magnetic signal modulation	L-/D-aspartic acid-modified cobalt hydroxide nanoparticles; chiral magnetic nanostructures	ROS-responsive imaging, tumor microenvironment analysis, enantioselective cellular uptake	[169,179,180]
FL/Chiroptical	Chiral interactions regulate fluorescence resonance energy transfer (FRET), circularly polarized luminescence (CPL), and circular dichroism (CD)	UCNP-based hybrid nanoprobes; Au–UCNP heterostructures; chiral nanopyramids	Intracellular imaging, ROS sensing, microRNA detection, bacterial identification	[144,176,177,181,182]
PAI	Chiral plasmonic assemblies improve optical absorption and photothermal conversion	DNA-guided Au–Ag nanoassemblies; L-/D-cysteine-functionalized plasmonic nanoparticles	Deep-tissue tumor imaging, photoacoustic-guided phototherapy, real-time treatment monitoring	[170]
Biosensing/Imaging	Chirality improves molecular selectivity through CD, plasmonic coupling, and FRET	Cysteine-capped quantum dots; chiral Co(OH)_2_ nanostructures; chiral triangular nanorings	Enantiomer recognition, biomarker detection, amyloid-β and α-synuclein sensing	[169,183,184]
SERS	Handedness-dependent plasmonic near fields produce distinct Raman enhancement for enantiomers, enabling sensitive chiral discrimination.	Amino acid capped nanoparticles, template-assisted synthesis, Chiral nanotubes, helicoids, nanoring, nanofilms	Enantiomer recognition, biomarker detection, amyloid sensing	[64,128,185]
Chiral membranes/nanochannels	Chiral interfaces regulate selective molecular transport and separation	Cu_2−x_Se/AAO membranes; Fe_3_O_4_ nanofilms; β-cyclodextrin-modified COF membranes	Enantioselective transport, drug separation, membrane-based biosensing	[186,187,188,189]

**Table 4:** Applications of chiral plasmonic materials for neuro- and age-related diseases.

Neurological Disorder/Biological Target	Representative Chiral Nanoplatform	Chirality-Associated Advantages	Primary Therapeutic Mechanism	Key Therapeutic Benefit	Ref
Alzheimer’s disease (Aβ pathology)	Glutathione-capped Au nanoparticles; D-/L-penicillamine-functionalized Fe–Cu–Se nanoparticles	Enantiomer-dependent interaction with Aβ aggregates, improved BBB penetration, enhanced ROS regulation	Disassembly of amyloid fibrils, ROS-mediated plaque clearance, neuroprotection	Lower amyloid burden and improved cognitive performance in AD models	[213,214,215]
Parkinson’s disease (α-synuclein pathology)	Chiral Cu_x_Co_γ_ superparticles; Mn_3_O_4_ nanorods; porous Cu_2_O nanoclusters	Chirality influences ROS scavenging efficiency and protein recognition	Suppression of α-synuclein aggregation, oxidative stress reduction, NIR-assisted fibril disruption	Restoration of motor function and neuronal protection in PD models	[205,211]
Neuroinflammation (Senescent microglia)	Antibody-conjugated chiral gold nanoparticles	Selective recognition and elimination of senescent microglial cells	Activation of Fas-mediated apoptosis and targeted clearance of dysfunctional microglia	Reduced neuroinflammation and improved cognitive function	[206]
Neural regeneration	Chiral Au nanofilms; CPL-responsive DNA nanoassemblies	Chirality and circularly polarized light promote neuronal adhesion and differentiation	Mechanical stimulation and activation of neuronal signaling pathways	Accelerated neural stem cell differentiation and enhanced tissue regeneration	[207,208]
Cellular senescence and aging	Chiral core–shell nanorods; Cu_x_Co_γ_ nanoparticles	D-enantiomer exhibits superior cellular uptake and senolytic performance	Mitochondrial dysfunction induction, ROS generation, caspase activation	Efficient removal of senescent cells and improvement of age-related tissue function	[210]
Photoresponsive senolytic theranostics	Chiral UCNP–AuNP–DNA tetrahedral nanostructures	Chirality enhances targeting accuracy and phototherapeutic responsiveness	NIR-triggered DNA cleavage, controlled granzyme B release, FRET-guided imaging	Simultaneous imaging and selective elimination of senescent cells	[212]

## Data Availability

No new data were created or analyzed in this study. Data sharing is not applicable to this article.
